# Artificial Neural Networks, Sequence-to-Sequence LSTMs, and Exogenous Variables as Analytical Tools for NO_2_ (Air Pollution) Forecasting: A Case Study in the Bay of Algeciras (Spain)

**DOI:** 10.3390/s21051770

**Published:** 2021-03-04

**Authors:** Javier González-Enrique, Juan Jesús Ruiz-Aguilar, José Antonio Moscoso-López, Daniel Urda, Lipika Deka, Ignacio J. Turias

**Affiliations:** 1 Intelligent Modelling of Systems Research Group (MIS), Department of Computer Science Engineering, Polytechnic School of Engineering, University of Cádiz, 11204 Algeciras, Spain; ignacio.turias@uca.es; 2 Intelligent Modelling of Systems Research Group (MIS), Department of Industrial and Civil Engineering, Polytechnic School of Engineering, University of Cádiz, 11204 Algeciras, Spain; juanjesus.ruiz@uca.es (J.J.R.-A.); joseantonio.moscoso@uca.es (J.A.M.-L.); 3 Grupo de Inteligencia Computacional Aplicada (GICAP), Departamento de Ingeniería Informática, Escuela Politécnica Superior, Universidad de Burgos, Av. Cantabria s/n, 09006 Burgos, Spain; durda@ubu.es; 4 The De Montfort University Interdisciplinary Group in Intelligent Transport Systems (DIGITS), Department of Computer Science and Informatics, De Montfort University, Leicester LE1 9BH, UK; lipika.deka@dmu.ac.uk

**Keywords:** forecasting, feature selection, air pollution, nitrogen dioxide, artificial neural networks, LSTMs, exogenous variables, deep learning, time series

## Abstract

This study aims to produce accurate predictions of the NO_2_ concentrations at a specific station of a monitoring network located in the Bay of Algeciras (Spain). Artificial neural networks (ANNs) and sequence-to-sequence long short-term memory networks (LSTMs) were used to create the forecasting models. Additionally, a new prediction method was proposed combining LSTMs using a rolling window scheme with a cross-validation procedure for time series (LSTM-CVT). Two different strategies were followed regarding the input variables: using NO_2_ from the station or employing NO_2_ and other pollutants data from any station of the network plus meteorological variables. The ANN and LSTM-CVT exogenous models used lagged datasets of different window sizes. Several feature ranking methods were used to select the top lagged variables and include them in the final exogenous datasets. Prediction horizons of *t* + 1, *t* + 4 and *t* + 8 were employed. The exogenous variables inclusion enhanced the model’s performance, especially for *t* + 4 (*ρ* ≈ 0.68 to *ρ* ≈ 0.74) and *t* + 8 (*ρ* ≈ 0.59 to *ρ* ≈ 0.66). The proposed LSTM-CVT method delivered promising results as the best performing models per prediction horizon employed this new methodology. Additionally, per each parameter combination, it obtained lower error values than ANNs in 85% of the cases.

## 1. Introduction

Nowadays, air pollution represents one of the main problems that affect the population’s quality of living, especially in densely populated areas. Low air quality can produce very harmful effects on human health, particularly on children and senior citizens [1,2]. Additionally, it also generates a sizable economic impact due to the increase in the cost of healthcare services.

Among air pollutants, nitrogen dioxide (NO_2_) generates a great deal of concern as it is considered a critical factor for air quality demise in urban areas [3]. This toxic gas is highly corrosive, very reactive, and possesses an intense irritating capacity [4]. NO_2_ origins are manifold: it is linked with traffic emissions and industrial operations, including combustion processes [5]. However, it is mainly a secondary pollutant, and its primary source can be found in the oxidation reactions between nitrogen oxides (NO) and ozone (O_3_) in the atmosphere [6]. The adverse effects of exposure to nitrogen dioxide include several diseases, such as bronchitis or pneumonia [7]. Its long-term impact on mortality is as remarkable as the effect produced by particulate matter [8]. Additionally, it has a significant role in generating photochemical smog acid rain [9]. 

Considering all the harmful effects that nitrogen dioxide may produce, it becomes essential to create accurate models to determine its future concentrations. Previous studies have addressed this purpose using two main approaches: deterministic approaches and statistical prediction. The deterministic approach employs mathematical formulations and the simulation of various physical and chemical processes, such as emission models, to predict airborne pollutants [10,11]. On the other hand, the statistical prediction approach creates statistical models based on historical data [12]. Unlike deterministic models, statistical techniques are not based on understanding the processes that regulate the change mechanism of pollutant concentrations. They are centered on discovering relations among historical data. Once found, these correlations are applied to the forecasting of future pollution levels. This statistical approach has been recognized as a viable alternative to the deterministic methods and, according to Catalano and Galatioto [13], can deliver better-performing models in short-term air pollutant concentrations. However, statistical methods are based on the assumption that the relations between variables are linear [14]. The irruption of machine learning (ML) techniques made possible the creation of models that could detect and capture non-linear relationships between variables. As a result, ML methods have been widely adopted by researchers for air quality prediction.

Several works devoted to NO_2_ time series forecasting using ML models can be found in the scientific literature in the last two decades. We can cite the work of Gardner and Dorling [15], who addressed the modeling of hourly NO_2_ concentrations using artificial neural networks (ANNs) in conjunction with meteorological data. Their results revealed how the proposed approach outperformed regression-based models. Another interesting study was undertaken by Kolehmainen et al. [16], where ANNs were employed to predict NO_2_ concentrations in Stockholm (Sweden). The authors obtained remarkable results using average NO_2_ values and several meteorological variables to feed the models. Viotti et al. [17] used ANNs for short and middle long-term forecasting of several pollutants, including NO_2_. Models exhibited excellent performances with a 1-h ahead prediction horizon. As the prediction horizon increased, the model’s performance decreased but was still better than deterministic models. Kukkonen et al. [18] evaluated the ANN model’s performance compared to other linear and deterministic models. Results brought to light how the neural network models provided better performances than the rest of the techniques tested. Aguirre-Basurko et al. [19] predicted O_3_ and NO_2_ in Bilbao (Spain). The authors compared ANN and multiple linear regression models using traffic data and meteorological variables as exogenous inputs in their study. Models were tested in several prediction horizons from *t* + 1 to *t* + 8, and ANN models showed the best performances in nearly all the proposed cases. Kumar and Jain [20] utilized an autoregressive moving average (ARIMA) approach to forecasting O_3_, NO, NO_2_, and CO with satisfactory results. Rahman et al. [21] compared ARIMA, fuzzy logic models, and ANN models to forecast the Air Pollution Index (API) in Malaysia. The API prediction implies predicting five pollutant concentrations: PM_10_, O_3_, CO_2_, SO_2_, and NO_2_. Results showed how ANN models gave the smallest forecasting errors. Bai et al. [22] utilized ANNs in conjunction with wavelet decomposition techniques to predict several pollutants, including NO_2_. The prediction horizon was set to 24 h, and results showed how the combined approach produced better results than standard ANNs. Finally, Van Roode et al. [23] proposed a hybrid model to forecast the NO_2_ concentration values with a one-hour prediction horizon in the Bay of Algeciras area (Spain). The authors employed LASSO to predict the linear part of the time series and ANN models to predict the residuals in a two-stage approach. The results confirmed that the proposed hybrid approach presented better performances than any of the particular methods employed.

Among machine learning methods, deep learning (DL) techniques have gained tremendous popularity in recent years. DL uses denser artificial neural networks combined with sequential layers and larger datasets than traditional machine learning methods. Long short-term memory networks (LSTMs) are recurrent neural networks specially designed for supervised time series learning [24]. Several studies have employed LSTMs to forecast pollutants in the scientific literature. We can cite the work of Kök et al. [25], where LSTMs and support vector regression (SVR) models were used to predict NO_2_ and O_3_ with a *t* + 1 horizon. Results showed how the LSTM model outperformed the SVR model. Another interesting study was undertaken by Pardo and Malpica [26], who proposed different LSTM models to predict NO_2_ levels for *t* + 8, *t* + 16 and *t* + 24 prediction horizons in Madrid (Spain). Finally, Rao et al. [27] compared LSTM based recurrent neural networks and SVR applied to air quality prediction. The results showed how the LSTM approach obtained better forecasting performances than the remaining method employed for all the pollutants considered.

Despite not explicitly being devoted to nitrogen dioxide forecasting, there are two interesting works worth mentioning. Kim et al. [28] developed a system to obtain daily PM_10_ and PM_2.5_ predictions in South Korea. In this work, the performances of LSTMs and chemical transport model simulations (more specifically, the Community Multiscale Air Quality (CMAQ) model) were compared. Different meteorological variables and several pollutants data (particulate matter, SO_2_, NO_2_ and NO_3_) were employed as input variables of the LSTM models. Results showed how LSTMs were able to outperform the CMAQ predictions in most of the cases considered. In the case of the study carried out by Carnevale et al. [29], a system to predict air quality in Milan (Italy) was proposed. This study was focused on obtaining up to 2 days ahead PM_10_ and ozone concentration predictions. A two-stage procedure was followed. In the first stage, neural network predictions were obtained for a monitoring network station located in the study area using exogenous variables. In the second stage, the forecasts obtained at each station were interpolated using the cokriging technique. Additionally, a deterministic chemical transport model was also included as a secondary variable. The proposed methodology provided satisfactory results and constituted a reliable way to give the decision-makers air quality forecasting.

**Figure 1:**
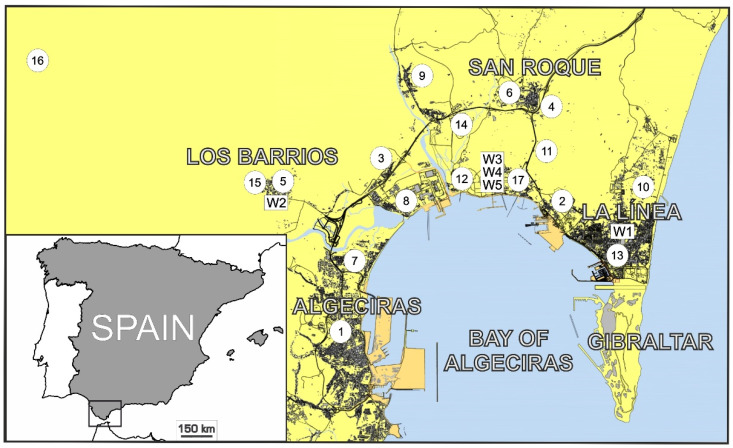
Location of the monitoring stations in the Bay of Algeciras.

In the present study, ANNs and sequence-to-sequence LSTMs models are developed to forecast NO_2_ concentrations in a specific station of a monitoring network located in the Bay of Algeciras area (Spain). The selected station is located in Algeciras, the study area’s principal city (see Figure 1). The primary goal is to build accurate statistical models to predict NO_2_ levels with *t* + 1, *t* + 4, and *t* + 8 prediction horizons. Two different approaches were followed to create the forecasting models. Only the NO_2_ data from the selected station were employed to feed the models in the first approach. In the second approach, exogenous variables were added to the set of predictor variables. In that sense, NO_2_ data from the network’s remaining stations, data from other pollutants (NO_x_, SO_2_, O_3_) from EPS Algeciras and other stations, and several meteorological variables were included (see Table 1). Based on the previously mentioned techniques, ANNs, standard sequence-to-sequence LSTMs, and LSTMs using a rolling window scheme in conjunction with a cross-validation procedure for time series (LSTM-CVT) were designed in both approaches. Finally, the obtained results were statistically analyzed and compared to determine the best performing model.

The rest of this paper is organized as follows. Section 2 details the study area and the data used. The modeling methods and feature ranking techniques used in this work are depicted in Section 3. Section 4 describes the experimental design. Section 5 discusses the results. Finally, the conclusions are presented in Section 6.

## 2. Data and Area Description

The Bay of Algeciras is a densely populated and heavily industrialized region situated in the south of Spain. The total population of this region in 2020 is estimated at 300,000 inhabitants [30]. It contains an oil refinery, a coal-fired power plant, a large petrochemical industry cluster, and one of the leading stainless-steel factories in Europe. 

As stated in the Introduction section, this work aims to predict the NO_2_ concentration levels with different time horizons in a monitoring network’s specific monitoring station. This station is EPS Algeciras (see Figure 1). It is located in Algeciras, the study area’s most populous city. With more than 120,000 inhabitants, its air quality is severely affected by the neighboring industries’ pollutant emissions. Additionally, the Port of Algeciras Bay can be found between the top 5 ship-trading ports in Europe. The high number of import and export operations held in this port implies high numbers of heavy vehicles and vessels every year. Combustion processes related to industrial activities and dense traffic episodes favor NO_2_ emissions, producing a very complicated pollution scenario (15 × 15 km^2^).

As was previously indicated, NO_2_ is one of the main factors of air quality decrease in urban areas. Therefore, having accurate models to predict its forthcoming concentrations becomes a critical task for environmental and governmental agencies. The proposed models can constitute a useful set of tools to predict exceedance episodes and take the corresponding corrective measures to avoid them. Additionally, the techniques presented in this article can also be applied to improve other pollutants’ predictions. These improved values can also help enhance the Air Quality Index’s [31] forecasts for the area of study. 

The data used in this work was measured by an air monitoring network deployed in the Bay of Algeciras area. It contains 17 monitoring stations and five weather stations. These weather stations are located in Los Barrios (W1), La Línea (W2), and a refinery property of the CEPSA company in different heights (W3 at 10 m, W4 at 60 m, and W5 at 15 m). Figure 1 shows the position of the stations in the study area. 

The database contains records of NO_2_, NO_x_, SO_2_, and O_3_ average hourly concentrations from January 2010 to October 2015. Several meteorological variables, measured hourly at the mentioned weather stations for the same period, are also included. The Andalusian Environmental Agency kindly provided all these measures. The complete list of variables included in the database is shown in Table 1.

Table 2 details the correspondence between the codes used in Figure 1 and the monitoring and weather stations. In this table, the pollutants and meteorological variables measured at each station are also indicated. It is important to note that not all pollutants are measured in all the monitoring stations. 

The database was preprocessed to eliminate possible outlier values and inaccurate measures caused by instrumental errors. After that, a process to impute this database’s missing values was applied using artificial neural networks as the estimation method.

## 3. Methods

Different models have been created to predict the NO_2_ level concentrations in this study. Two main forecasting techniques were employed: artificial neural networks and sequence-to-sequence LSTMs. Additionally, a new methodology was proposed based on the LSTM technique previously mentioned: LSTM-CVT. A concise description of these forecasting techniques is presented in Section 3.1.

The input data for ANNs and the input sequence for LSTM-CVT have been obtained using a rolling window method. The procedure to build the new lagged variables dataset is described in Section 3.2. Additionally, the ANN and LSTM-CVT models employ a cross-validation method for time series described in Section 3.3. 

As was stated in the Introduction section, two different approaches have been compared in this study according to the type of input variables used. In the second one, the use of exogenous variables implies a group of lagged variables for ANN and LSTM-CVT models equal to the selected window size multiplied by the total number of input variables. Section 3.4 describes the feature ranking methods employed in this work to selects the best among these lagged variables.

### 3.1. Forecasting Techniques

#### 3.1.1. Artificial Neural Networks

Artificial neural networks are a branch of machine learning techniques inspired by how the human brain operates to recognize the underlying relationships in a data set. They are made of several interconnected non-linear processing elements, called neurons. These neurons are arranged in layers, which are linked by connections, called synapses weights. ANNs can detect and determine non-linear relationships between variables. They can act as non-linear functions that map predictors and dependent variables.

Feedforward multilayer perceptron trained by backpropagation (BPNN) [32] is the most commonly used neural network type. Its architecture includes an input layer, one or more hidden layers, and an output layer. The networks are organized in fully connected layers. Their learning process is based on information going forward to the following layers and errors being propagated backward, in a process called backpropagation. According to Hornik et al. [33], feedforward neural networks with a single hidden layer can approach any function if they are correctly trained and contain sufficient hidden neurons. Hence, they are considered a type of universal approximators. BPNNs can be applied either to regression or classification problems [34] where no a priori knowledge is known about the relevance of the input variables. These characteristics make them an adequate method to solve different problems of high complexity, especially non-linear mappings [35]. However, ANNs also present some disadvantages: the inexistence of a standard approach to determine the number of hidden units and possible overfitting affecting the models.

In this work, BPNNs models have been trained using the scaled conjugate gradient backpropagation algorithm [36] to build NO_2_ forecasting models. The generalization capability of the mentioned models constitutes a crucial matter. Generalization can be defined as the network’s ability to produce good results for unseen new data [34]. Therefore, the reduction of the generalization error becomes essential to obtain accurate prediction models. In that sense, the early stopping technique [35,37] was employed in the models’ training phase to reduce overfitting and avoid generalization issues. The optimal number of hidden neurons was settled by a resampling procedure using 5-fold time-series cross-validation (see Section 3.3). The authors have successfully applied a similar resampling procedure in previous works [38,39,40,41,42], but in this case, it has been modified to time series prediction.

#### 3.1.2. Long Short-Term Memory Networks

Long short-term memory networks are a type of recurrent neural network proposed by Hochreiter and Schmidhuber [43]. Some years later, they were greatly enhanced by Gers et al. [24] by including the fundamental forget gate concept. Standard RNNs can learn past temporal patterns and correlations but are limited when dealing with long-term dependencies in sequences because of the vanishing gradient problem [44,45]. LSTMs overcome this situation by including a special type of unit called memory blocks in their architecture. These units allow LSTMs to decide which meaningful information must be retained, learn long-term dependencies and capture contextual information from data, making them especially suitable for time series prediction [46]. 

**Figure 2:**
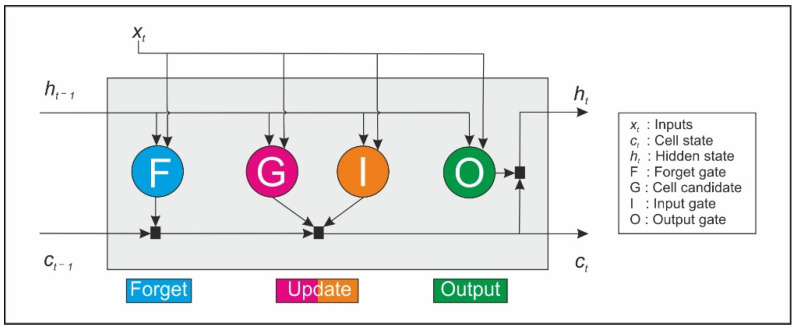
Architecture of a memory block with a single cell.

The basic architecture of the LSTM models includes an input layer, a recurrent hidden layer (LSTM layer) containing the memory blocks (also called neurons), and an output layer. One or more self-connected memory cells are included in each memory block. Additionally, three multiplicative units are also contained inside the memory blocks: input, output and forget gates. These gates provide read, write and reset capabilities, respectively, to the memory block. Additionally, they enable LSTMs to decide which meaningful information must be retained and which not relevant information must be discarded. Therefore, they allow the control of information flow and permit the memory cell to store long-term-dependencies. A schematic representation of a memory block with a single cell is shown in Figure 2.

**Figure 3:**
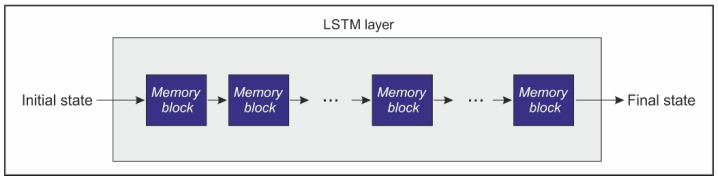
Schematic representation of the long short-term memory network (LSTM) layer structure.

A typical LSTM includes several memory blocks or neurons in the LSTM layer arranged in a chain-like structure. A schematic representation of this structure is depicted in Figure 3. 

The cell state and the hidden state are the main properties of this type of network. These properties are sent forward from one memory block to the next one. At time *t*, the hidden state (*h_t_*) represents the LSTM layer’s output for this specific time step. The cell state constitutes the memory that contains the information learning from the previous timestamps. Data can be added or eliminated from this memory employing the gates. The forget gate F controls the connection of the input (*x_t_*) and the output of the previous block (hidden state *h_t_*_−1_) with the cell state received from the previous block (*c_t_*_−1_). Then it selects which values from *c_t_*_−1_ must be retained and which ones discarded. After that, the input gate decides which values of the cell state should be updated. The cell candidate then creates a vector of new candidate values, and the cell state is updated, producing *c_t_*. Finally, the outputs of the memory block are calculated in the output gate. All this process can be formulated as described in Equations (1)–(5) [47].

(1) $$i_{t}=\delta \left(W_{xi}x_{t}+W_{hi}h_{t-1}+W_{ci}⊙c_{t-1}+b_{i}\right)$$

(2) $$f_{t}=\delta \left(W_{xf}x_{t}+W_{hf}h_{t-1}+W_{cf}⊙c_{t-1}+b_{f}\right),$$

(3) $$c_{t}=f_{t}⊙c_{t-1}+i_{t}⊙\tan h(W_{xc}x_{t}+W_{hc}h_{t-1}+b_{c})$$

(4) $$o_{t}=\delta \left(W_{xo}x_{t}+W_{ho}h_{t-1}+W_{co}⊙c_{t}+b_{o}\right)$$

(5) $$h_{t}=o_{t}⊙\tan h(c_{t}),$$

where *f_t_*, *i_t_* and *o_t_* indicate the state of the forget gate, the input gate and the output gate at time t, respectively. Additionally, *h_t_* refers to the hidden state and *c_t_* stands for the cell state. *W_xi_*, *W_hi_*, *W_ci_*, *W_xf_*, *W_hf_*, *W_cf_*, *W_xc_*, *W_hc_*, *W_xo_*, *W_ho_* and *W_co_*, correspond to the trainable parameters. The operator ⊙ denotes the Hadamard product, and the bias terms are represented *b_i_*, *b_f_*, *b_c_* and *b_o_*. Finally, *δ* corresponds to the sigmoid function and tanh indicates the hyperbolic tangent function, which are expressed in Equations (6) and (7), respectively.

(6) $$\delta \left(x\right)={\left(1+e^{-x}\right)}^{-1}$$

(7) $$\tan h\left(x\right)=\frac{e^{x}-e^{-x}}{e^{x}+e^{-x}}$$

The function of the memory blocks is similar to the neurons in shallow neural networks. In that sense, in the rest of the paper, these memory blocks are referred to as LSTM neurons.

### 3.2. Lagged Dataset Creation

The time series were transformed into a dataset suitable for the ANN and LSMT-CVT models using a rolling window approach. Autoregressive window sizes of 24, 48 and 72 h were employed. The lagged dataset creation follows a different procedure depending on the type of input variables used: univariate time series and multivariate time series.

In the first case, only the hourly NO_2_ measures from the selected station were used. New lagged variables were built based on samples of consecutive observations [48]. Thus, the datasets were defined as $D_{k,ws}={\left\{x_{ws}{}_{i},\;y_{k}{}_{i}\right\}}_{i=1}^{T}$ where *T* indicates the number of samples, *k* is the prediction horizon and *ws* corresponds to the window size. Each *i*-th sample (row of the dataset) was defined as an input vector $x_{i}=\left\{x_{i}\left(t\right),\ldots ,x_{i}\left(t-\left(ws-1\right)\right)\right\}$ concatenated to its corresponding output value $y_{i}=\left(t+k\right)$. These new lagged datasets were split into a subset for training and a second subset for testing. The first one included the first 70% of the records and was used to train the models and determine their hyperparameters. The remaining 30% was used as the test subset. In this sense, the models’ performance was tested using unseen data from this subset. 

In the second case, data from exogenous features were also included in the group of initial inputs. These time series were also transformed into new lagged datasets appropriate to feed the models using the same window sizes as the previous case. The following steps summarize this process:

1. For each initial variable *v_j_*, lagged variables (column vectors) were built in a similar way to the univariate case: {$v_{j}\left(t\right),\ldots ,v_{j}\left(t-\left(ws-1\right)\right)$}.
2. As a second step, the group of potential input variables *P_ws_* was created including all the previously created lagged variables *P_ws_* = {$(v_{1}\left(t\right),\ldots ,v_{1}\left(t-\left(ws-1\right)\right)),\ldots ,\left(v_{j}\left(t\right),\ldots ,v_{j}\left(t-\left(ws-1\right)\right)\right)$} where *j* indicates the total number of initial variables (77 variables, see Table 1).
3. Then, new datasets were created, including the potential group of variables and the output variable. These datasets were split into training (first 70% records) and test subsets (ending 30% records).
4. As a next step, several feature ranking methods were applied to the elements of *P_ws_* in the training subset. The feature ranking methods applied were: mutual information, mutual information using the minimum-redundancy-maximum-relevance algorithm, Spearman’s rank correlation, a modified version of the previously mentioned algorithm using Spearman’s rank correlation, and maximal information coefficient (see Section 3.4). The objective was to select the most relevant among the lagged variable concerning the output variable y=(t+k). The selected lagged variables of the training set were included in the $STrain_{k,ws,fr,per}$ set, where *fr* indicates the feature ranking method applied and *per* corresponds to the percentage of lagged features selected. Thus, only a small portion of the potential lagged variables was chosen to be finally included as a column in the dataset (the top 5%, top 10% and top 15% variables). Once the ranking and selection process was completed, the same selection criteria were applied to the test set, obtaining the $STest_{k,ws,fr,per}$ set.

As the final step, the final training and test datasets were defined as $DTrain_{k,ws,fr,per}={\left\{STrain_{k,ws,fr,per}{}_{i},\;y_{k}{}_{i}\right\}}_{i=1}^{T}$ and $DTest_{k,ws,fr,per}={\left\{STest_{k,ws,fr,per}{}_{i},\;y_{k}{}_{i}\right\}}_{i=1}^{T}$. Each *i*-th sample (row) of these datasets was defined as an input vector which included the selected lagged variables, as well as its corresponding output value $y_{i}=\left(t+k\right).$ Consequently, the datasets included the selected lagged variables in separate columns and the output variable *y* occupying the final column.

### 3.3. Time Series Cross-Validation 

Cross-validation is a widespread technique in machine learning. However, as was stated by Bergmeir and Benítez [49], there exist some problems regarding dependencies and temporal evolutionary effects within the time-series data. Traditional cross-validation methods do not adequately address these issues. In the *k*-fold cross-validation method [50,51], all the available training data is randomly divided into *k* folds. The training procedure is then performed using *k* − 1, folds, and the error is obtained using the remaining fold as the test set. This procedure is repeated *k* times so that each fold is used as the test set once. Finally, the error estimate is obtained as the average error rate on test examples.

Bergmeir and Benítez [49] recommend using a blocked cross-validation method for time series forecasting to overcome these shortcomings. This procedure follows the same steps as the *k*-fold cross-validation method, but data is partitioned into *k* sequential folds respecting the temporal order. Additionally, dependent values between the training and test sets must be removed. In this sense, an amount of lagged values equal to the window size used is removed from the borders where the training and the test sets meet. 

**Figure 4:**
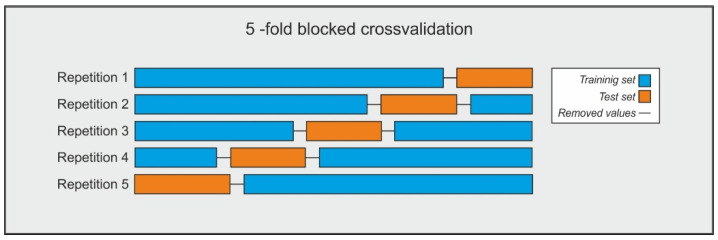
Scheme of the 5-fold blocked cross-validation followed in this work.

In this work, a 5-fold blocked cross volition method was followed. This method allowed us to determine the hyperparameters of the ANN y LSTM-CVT models (in this last case, in conjunction with the Bayesian optimization technique, see Section 4). A representation of this scheme is presented in Figure 4 [52]. 

### 3.4. Feature Ranking Methods

The feature ranking methods employed to select the most meaningful lag variables in the lagged dataset creation are briefly presented in this Section.

#### 3.4.1. Mutual Information

Mutual information (MI) [53] measures the amount of information that one vector contains about a second vector. It can determine the grade of dependency between variables. MI can be defined by Equation (8).

(8) $$MI\left(x,y\right)=\iint p\left(x,y\right)\;\log\frac{p\left(x,y\right)}{p\left(x\right)\cdot p\left(y\right)}\;dxdy,$$

where *x* and *y* are two continuous random vectors, is their joint probability density and *p*(*x*) and *p*(*y*) are their marginal probability density. Equation (8) can be reformulated to obtain Equation (11) utilizing entropy (Equation (9)) and conditional entropy (Equation (10)).

(9) $$H\left(x\right)=-\int_{S}p\left(x\right)\log p\left(x\right)dx,$$

where *S* is the set of the random vector with *p*(*x*) > 0.

(10) $$H\left(y|x\right)=\iint p\left(x,y\right)\log\frac{p\left(x\right)}{p\left(x,y\right)}dxdy,$$

where 0<H(y|x)<H(x).

(11) MI(x,y)=H(y)−H(y|x).

The ITE Toolbox [54] was employed to calculate *MI* throughout the present manuscript.

#### 3.4.2. Maximal Information Coefficient 

Proposed by Reshef et al. [55], the maximal information coefficient (MIC) can reveal linear and non-linear relationships between variables and measure the strength of the relationship between them. Given two vectors, *x* and *y*, their MIC can be obtained employing Equation (12) [56].

(12) $$MIC\left(x,y\right)=\max\left\{MI\left(x,y\right)/\log_{2}\min\left\{n_{x},n_{y}\right\}\right\},$$

where *MI*(*x,y*) indicates the mutual information between *x* and *y*, and *n_x_,n_y_* corresponds to the number of bins dividing *x* and *y*. This study’s MIC values were obtained through the Minepy package for Matlab [57].

#### 3.4.3. Spearman’s Rank Correlation Coefficient

Spearman’s rank correlation (SRC) assesses the monotonic relationship’s strength and direction between two variables. This non-parametric measure is calculated operating on the data ranks, with values ranging from [−1,1]. Given two variables *x* and *y*, the Spearman’s rank correlation between them can be calculated using Equation (13).

(13) $$r_{x,y}=\frac{\sum_{i=1}^{n}\left\{\left(x_{i}-\bar{x}\right)\cdot \left(y_{i}-\bar{y}\right)\right\}}{\sqrt{\sum_{i=1}^{n}{\left(x_{i}-\bar{x}\right)}^{2}\cdot \sum_{i=1}^{n}{\left(y_{i}-\bar{y}\right)}^{2}}}.$$

#### 3.4.4. Minimum-Redundancy-Maximum-Relevance

Minimum-redundancy-maximum relevance (mRMR) [58] is a feature ranking algorithm that penalizes redundant features. This algorithm aims to rank the input variables according to their balance between having maximum relevance with the target variable and minimum redundancy with the remaining features. Relevancies and redundancies are calculated using mutual information. The pseudocode of the mRMR algorithm [59], modified to be used in regression problems, is shown in Algorithm A1 of Appendix A. 

In this work, MI, MIC, SRC and mRMR were used to select the most relevant variables when exogenous variables were employed (see Section 3.2). Additionally, the mRMR algorithm was also modified so that Spearman’s rank correlation was used to calculate relevancies and redundancies between variables (mRMR-SRC). Consequently, the relevance term (line 2) and the redundancy term (line 5) of the algorithm were modified, as shown in Algorithm A2 of Appendix A.

## 4. Experimental Procedure

In this study, ANNs, sequence-to-sequence LSTM and the proposed LSTM-CVT method were used to predict the NO_2_ concentration levels in the EPS Algeciras monitoring station (see Table 2 and Figure 1). The following prediction horizons were used to create the forecasting models: *t* + 1, *t* + 4, and *t* + 8. Additionally, two different approaches were followed in the model’s creation regarding the initial input data used: using only the NO_2_ data from the EPS Algeciras station (univariate dataset) or using all the available data (exogenous dataset). This second possibility includes all the 77 variables listed in Table 1 (NO_2_ and other pollutants (NO_x_, SO_2_, O_3_) from EPS and the remaining stations and several meteorological variables). As was mentioned in Section 2, the database included hourly measures from January 2010 to October 2015. As the first step for both approaches, all the dataset was preprocessed and standardized. 

The performance indexes utilized to evaluate the generalization capabilities of the models and their performance were the Pearson’s correlation coefficient (*ρ*), the mean squared error (*MSE*), the mean absolute error (*MAE*) [60] and the index of agreement (*d*). Lower values of *MSE* and *MAE* are associated with more accurate predictions, while higher values of *d* and *ρ* indicate higher performance levels of the models. Their corresponding definitions are shown in Equations (14)–(17).

(14) $$\rho =\frac{\sum_{i=1}^{N}\left(O_{i}-\bar{O}\right)\cdot \left(P_{i}-\bar{P}\right)}{\sqrt{\sum_{i=1}^{N}{\left(O_{i}-\bar{O}\right)}^{2}\cdot \sum_{i=1}^{N}{\left(P_{i}-\bar{P}\right)}^{2}}},$$

(15) $$MSE=\frac{1}{N}\;\sum_{i=1}^{N}{\left(P_{i}-O_{i}\right)}^{2},$$

(16) $$MAE=\frac{1}{N}\;\sum_{i=1}^{N}\left|P_{i}-O_{i}\right|,$$

(17) $$d=1-\frac{\sum_{i=1}^{N}{\left(P_{i}-O_{i}\right)}^{2}}{\sum_{i=1}^{N}{\left(\left|(P_{i}-\bar{O}\right|+\left|O_{i}-\bar{O}\right|\right)}^{2}},$$

where *P* indicates the model predicted values and *O* represents the observed values.

Table 3 summarizes the characteristics of the forecasting models employed in this paper. A detailed description of the experimental procedure followed in each case is presented in the following subsections. 

### 4.1. LSTM-UN and LSTM-EX Models

These models were built using sequence-to-sequence LSTMs. The sequence-to-sequence architecture employs an encoder-decoder structure to transform the inputs by an encoding procedure to a fixed dimensionality vector. This intermediate vector is then decoded to produce the final output sequence [61]. In this technique, minimal assumptions are made on the sequence structure, and the LSTM models map an input sequence of values corresponding to *T* time steps $x=\left(x_{1},\ldots ,x_{T}\right)$ to an output sequence of values $y=\left(y_{1},\ldots ,y_{T}\right)$.

The univariate and exogenous datasets were split into two disjoint training and testing subsets as a first step. The training subset included the first 70% of the records and was used to train the models and determine their hyperparameters. The remaining 30% was used as the test subset. In this sense, the models’ performance was tested using unseen data from this subset.

In the case of the LSTM-UN models, the univariate datasets were used. Input and output sequences were created for the training and test subsets. The output sequences were obtained from their corresponding input sequences with values shifted by *k* time steps, where *k* indicates the forecasting horizon (*t* + 1, *t* + 4 and *t* + 8 in this work). After that, the models were trained using the input and output sequences corresponding to the training subset. Bayesian optimization [62,63] was employed to select the optimal learning hyperparameters utilizing the bayesopt MATLAB function with 500 interactions. The root mean square error was the metric employed in this optimization process. The parameters used are shown in Table 4.

The Adam optimizer was employed to train the LSTM models, whose architecture is detailed in Table 5. A dropout layer [64] was added to the standard architecture used in sequence-to-sequence regression problems. This layer aims to prevent overfitting by randomly setting input elements to zero with a given probability. 

Then, the training phase’s best network was fed with the input sequence corresponding to the test subset. As a result, the NO_2_ predicted values were obtained. Finally, performance measures were assessed by comparing the test subset’s output sequence against these forecasted values.

**Figure 5:**
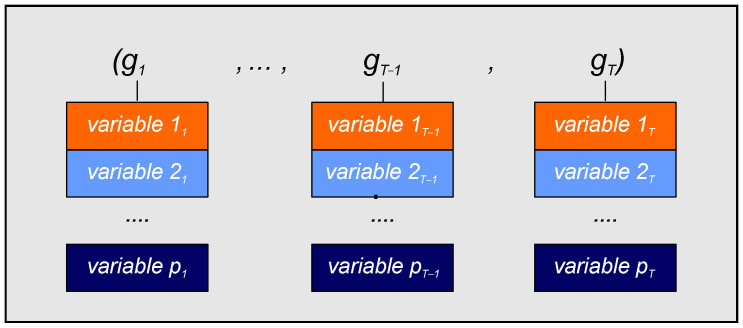
Schematic representation of the exogenous input sequence of time *T* time steps, where *p* indicades the total number of variables employed.

In the case of the LSTM-EX, the process followed is precisely the same as the LSTM-UN models, except for the sequences employed. Thus, the original input sequences corresponding to the training and test subsets had to be modified as these models used the exogenous datasets. Each element of a given original input sequence *x* was updated to include the new exogenous variables. As a result, an exogenous input sequence $g=\left(g_{1},\ldots ,g_{T}\right)$ was obtained. In this new sequence, every element was a column matrix, with $g_{j}\in {\mathbb{R}}^{px1}$ and *p* corresponding to the total number of variables used (see Table 1). A graphical representation of this exogenous sequence is presented in Figure 5.

### 4.2. ANN-UN and ANN-EX Models

The NO_2_ forecasting models using ANNs are illustrated in this subsection. In the first step, lagged training and test datasets were created for each case, as described in Section 3.2. BPNNs were trained using the lagged training dataset following a 5-fold fold cross-validation scheme for time series, as described in Section 3.3. This model’s architecture included a fully connected single hidden layer and several hidden units (1 to 25). The scaled conjugate gradient backpropagation algorithm was employed in conjunction with the early stopping technique. This process was repeated 20 times, and the average results were calculated and stored. Table 6 summarizes all the parameters used in the ANN models.

Additionally, a multi comparison procedure aimed to discover the simplest model without significant statistical differences with the best performing model was undertaken. As a first step, the Friedman test [65] was applied to the test repetitions previously stored. This test (non-parametric alternative to ANOVA) allowed us to determine if relevant differences were present between models built using a different number of hidden units. If differences were detected, models statistically equivalent to the best performing model were found employing the Bonferroni method [66]. Among them, the simplest model was finally selected according to Occam’s razor principle. 

After that, a final BPNN model was trained using the entire lagged training dataset. The number of hidden units used was the one determined in the previous step. Once trained, the inputs of the test lagged dataset were used to feed this model. As a result, the NO_2_ predicted values were obtained, and performance measures were calculated comparing predicted against measured values. This process was repeated 20 times, and the average results were calculated.

### 4.3. LSTM-CVT-UN and LSTM-CVT-EX Models

The proposed LSTM-CVT method employed sequence-to-sequence LSTMs. However, the input data sequences used did not comprise all the *T* time steps. In contrast, a rolling window approach was utilized to create lagged training and test datasets, following the procedure described in Section 3.2. 

In the case of the LSTM-CVT-UN, the univariate dataset was used to create the lagged training and test datasets. The same parameters (see Table 4) and network architecture (see Table 5) described in the case of the LSTM-UN were also employed in this case. The Adam optimizer was employed in the training process, and 500 interactions were used to determine the optimal hyperparameters through the Bayesian optimization algorithm. The average *MSE* was the metric employed in this optimization procedure.

Each of these interactions represents a different parameter combination. Per each of them, a 5-fold fold cross-validation scheme for time series (see Section 3.3) was applied to the lagged training dataset. Thus, this dataset was divided into five sequential folds: four of these folds acted as the training subset, while the reaming one served as the test subset. Additionally, an amount of lagged values equal to the window size was eliminated from zones where training and test subsets come together (see Figure 4). Then, the training subset’s input and output sequences were used to train the sequence-to-sequence LSTM models. Once the model was trained, it was fed by the input sequence of the test subset, and the *MSE* was calculated by comparing the predicted values against the output sequence of the test subset. This procedure was repeated five times until all the folds were employed once as the test subset. Finally, the average value of *MSE* was calculated.

After the optimal parameters were found, a sequence-to-sequence final LSTM model was trained using the entire lagged training dataset. Once trained, the input sequence of the test lagged dataset was used to feed this model. As a result, the NO_2_ predicted values were obtained. Performance measures were calculated comparing these values against the output sequence of the test lagged dataset. This process was repeated 20 times, and the average results were calculated.

In the LSTM-EX models, the procedure followed is the same as described in LSTM-UN models. However, as the exogenous datasets were used, the input sequences of their corresponding training and test lagged datasets had to be modified. This modification was performed as described for the LSTM-EX models case (see Section 4.1 and Figure 5). 

## 5. Results and Discussion

This section contains the results obtained in this study on forecasting the NO_2_ concentration in the EPS Algeciras monitoring station situated in the Bay of Algeciras area. All the calculations were carried out using MATLAB 2020a running on an Intel Xenon 6230 Gold workstation, equipped with 128 GB of RAM and an NVidia Titan RTX graphic card. 

The performance metrics depicted in this section correspond to the final models calculated using the test subset with the ending 30% of the database’s records. The models were built employing ANNs, sequence-to-sequence LSTMs and the novel LSTM-CVT method as the forecasting techniques. Prediction horizons of *t* + 1, *t* + 4 and *t* + 8 were established, and their performance was compared in two different scenarios depending on the dataset used (univariate or exogenous datasets, see Section 4).

In the ANN and LSTM-CVT models, different sizes of autoregressive windows were used (24, 48 and 72 h). In the models where an exogenous dataset was used, only the top 5%, 10% or 15% lagged variables were kept (see Section 3.2). This selection was made according to several feature ranking techniques: mutual information, mRMR, Spearman’s rank correlation, mRMR-SRC and MIC (see Section 3.4).

Table 7 shows the average performance for the top models per each prediction horizon. In this table, *ws* corresponds to the window size, *nh* is the number of units in the hidden layer (neurons), *DP* denotes the dropout probability, *MBS* is the minibatch size, *LR* corresponds to the learning rate, *L*2*R* is the level 2 regularization factor and *GD* is the gradient decay factor. In the exogenous datasets scenario, the top models per window size are presented. Additionally, Table A1, Table A2, Table A3, Table A4, Table A5 and Table A6 in Appendix A show the results obtained using the univariate dataset and the top models per window size using exogenous datasets. The complete list of models built using exogenous datasets is also presented in Table A7, Table A8 and Table A9 of the mentioned appendix.

**Figure 6:**
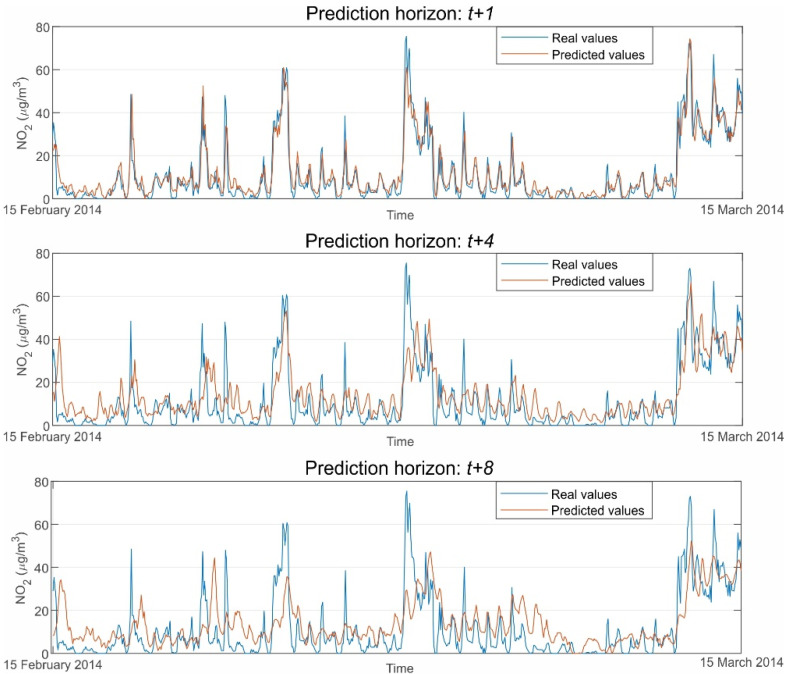
Observed vs. predicted values of NO_2_ hourly average concentrations for the top models of Table 7.

A first comparison of the results based on the prediction horizon shows how performance indices worsen as the forecast horizon grows. As further in the future, the prediction goes, the accuracy of the models lowers. Thus, the best performing models go from *ρ* ≈ 0.90 for *t* + 1 to *ρ* ≈ 0.66 for *t* + 8. A comparison between the top models for each prediction horizon of Table 7 is presented in Figure 6. In this figure, observed vs. predicted values of NO_2_ hourly average concentrations are depicted for the period between the 15 February 2014 and the 15 March 2014.

As can be seen, the fit and adjustment to the measured values are excellent for the best model of the *t* + 1 prediction horizon. However, the fit’s goodness decreases as the prediction horizons grow, confirming what was previously stated.

Another essential factor in this work is the possible influence of exogenous variables on the models’ performance. In light of the results, exogenous variables’ inclusion boosts the model’s forecasting performance, regardless of the forecasting technique used or the prediction horizon considered. Table 8 shows the perceptual changes in *ρ* and *MSE* of the models from Table A1, Table A2, Table A3, Table A4, Table A5 and Table A6 (see Appendix A).

As can be observed, exogenous variables produce a noticeable enhancement in all the cases considered. This improvement becomes greater for *t* + 4 and *t* + 8, especially for the Pearson’s correlation coefficient. An in-depth look at the results shows how the proposed LSTM-CVT-EX models lead the prediction horizon scenarios’ performance rankings. Additionally, the LSTM-CVT and ANN best-performing models provide better performance indexes than sequence-to-sequence LSTMs in all the proposed cases. This observation emphasizes the positive effect of the lagged dataset and the time series cross-validation on the LSTM-CVT models, which internally uses sequence-to-sequence LSTMs. 

**Figure 7:**
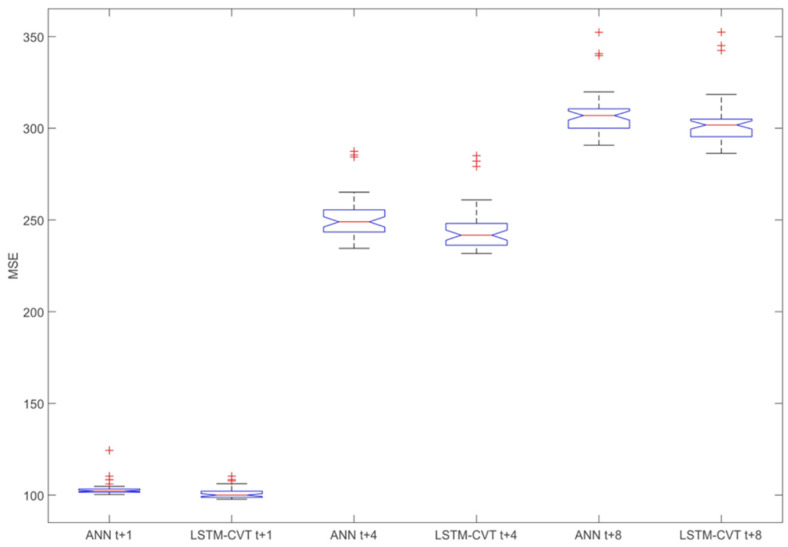
Comparison of the exogenous artificial neural network (ANN) and LSTM-CVT models according to their average *MSE* values for *t* + 1, *t* + 4 and *t* + 8 prediction horizons. In each case, all the possible windows size, feature ranking and percentage combinations are included.

The comparison of the LSTM-CVT and the ANNs models reveals that their performances are much closer than in the previous case. However, all the best performing models per prediction horizon are LSTM-CVT models. This fact can also be observed for each prediction horizon/window size combination presented in Table A1, Table A2, Table A3, Table A4, Table A5 and Table A6. Figure 7 depicts box-plot comparisons of these models for exogenous datasets and *t* + 1, *t* + 4 and *t* + 8 prediction horizons. For each case, their average *MSE* values have been compared, including all the possible windows size, feature ranking and percentage combinations considered in this work (see Appendix A for the complete list of cases for the exogenous datasets).

**Figure 8:**
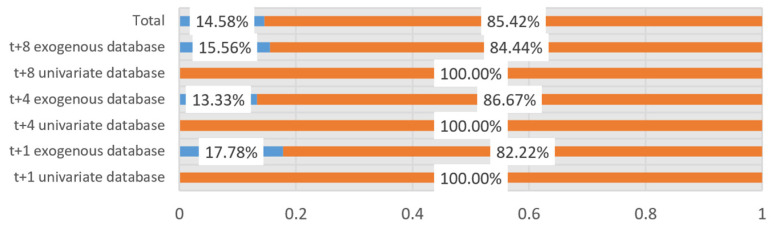
Comparison of the ANN and LSTM-CVT models using the same parameter configuration. The rate of parameter combinations where each technique provides better average *MSE* values are indicated.

Additionally, per each parameter combination (window size + feature ranking method + percentage), ANN and LSTM-VCT models have been compared. The rates of parameter combinations where each technique provides better average *MSE* values are presented in Figure 8. The representations in Figure 7 and Figure 8 confirm the forecasting capability of the LSTM-CVT method as it offers a lower average *MSE* than ANN models in the 85% of the total combinations considered.

**Figure 9:**
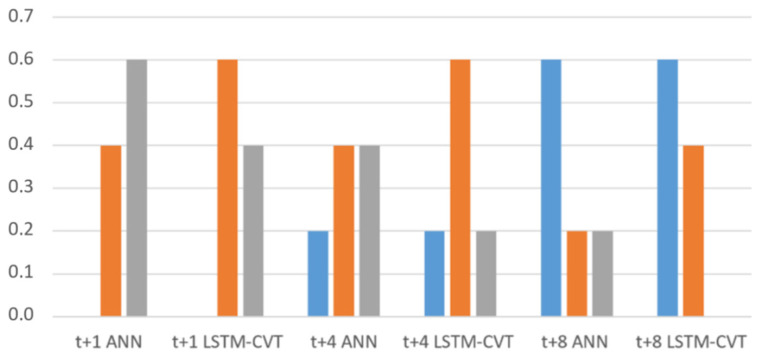
Window sizes used in the top 10% exogenous models.

**Figure 10:**
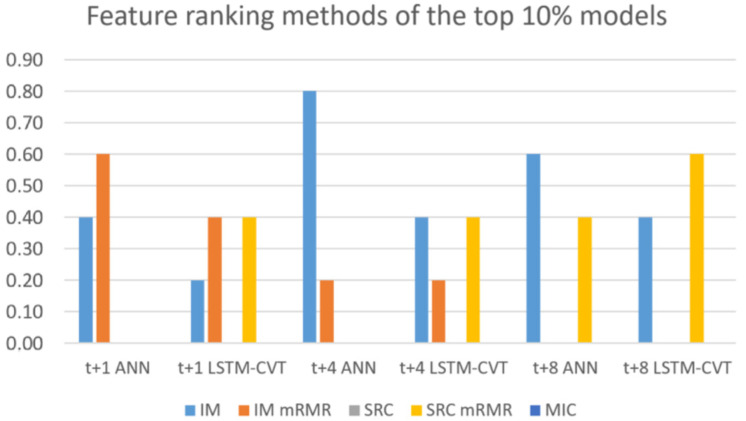
Feature ranking methods employed in the top 10% exogenous models.

**Figure 11:**
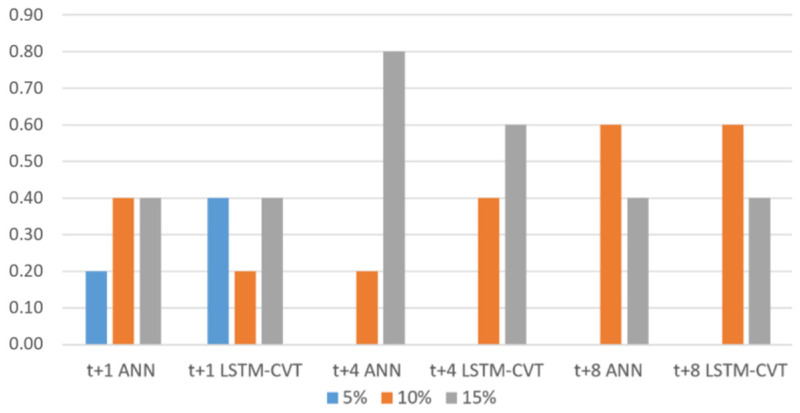
Percentage of lagged variables selected in the top 10% exogenous models.

Another interesting aspect is related to the window size, percentage of lagged variables selected used by the top-performing models. Figure 9, Figure 10 and Figure 11 depicts the usage rates of their possible values for these parameters by the top 10% performing models.

As shown in Figure 9, window sizes of 48 h are among the more employed, with an approximate usage of the 43% of the models considered. However, 72 and 24 h are also employed with use percentages of around 30%. The difference is that *t* + 1 models tend to use larger window sizes (48–72 h), while *t* + 8 models do the opposite (24 is the preferred window size in this prediction horizon).

Regarding the feature ranking techniques employed, it is essential to note the influence of these methods in the exogenous lagged dataset creation and, hence, the model’s future performance. Figure 10 shows how the top-performing models only use mutual information, mRMR and mRMR-SRC. In contrast, MIC and standard Spearman’s rank correlation are not employed by these top-performing models. On the one hand, mutual information is applied by around 50% of the models. A closed look to Figure 10 reveals that MI is especially significant in ANN models, while LSTM-CVT models use mRMR-SRC much more. Additionally, the use of mRMR decreases as the prediction horizon grows (it is not employed by any of the top-performing models in *t* + 8). 

Concerning the percentage of lagged variables used presented in Figure 11, the options of 15% and 10% are used in all the cases. Their use is especially remarkable in longer time horizons. Conversely, the 5% option is only used by *t* + 1 models that do not need as much information as *t* + 4 or *t* + 8 models to provide good forecasting results. 

## 6. Conclusions

This paper aims to produce accurate forecasting models to predict the NO_2_ concentration levels at the EPS Algeciras monitoring station in the Bay of Algeciras area, Spain. The forecasting techniques employed include ANNs, LSTMs and the newly proposed LSTM-CVT method. This method merges sequence-to-sequence LSTMs with a time-series cross-validation procedure and a rolling window approach to utilize lagged datasets. Additionally, a methodology used to feed standard sequence-to-sequence LSTMs with exogenous variables was also presented. Bayesian optimization was employed to automatically determine the optimal hyperparameters of the LSTM models, including LSTM-CVT.

Three different prediction horizons (*t* + 1, *t* + 4 and *t* + 8) were established to test the forecasting capabilities. Additionally, two different approaches were followed regarding the input data. On the one hand, the first option used a univariate dataset with just the hourly NO_2_ data measured at the EPS Algeciras monitoring station. On the other hand, the second approach added exogenous features, including NO_2_ data from different monitoring stations, other pollutants (SO_2_, NO_x_ and O_3_) from EPS and the remaining stations, and several meteorological variables. 

The procedure used to create the ANN and LSTM-CVT exogenous models includes creating lagged datasets with different window sizes (24, 48 and 72 h). The high number of features employed made it unfeasible to use all the lagged variables produced. Hence, several feature ranking methods were presented and used to select the top 5%, 10% and 15% lagged variables into the final exogenous datasets. Consequently, 45 window size/feature ranking/percentage combinations were arranged and tested per each prediction horizon (see Appendix A).

Exogenous datasets produced a noticeable enhancement in the model’s performance, especially for *t* + 4 (*ρ* ≈ 0.68 to *ρ* ≈ 0.74) and *t* + 8 (*ρ* ≈ 0.59 to *ρ* ≈ 0.66). In the case of the *t* + 1 horizon, results were closer (*ρ* ≈ 0.89 to *ρ* ≈ 0.90). These improvements are found no matter the prediction technique used (see Table A1, Table A2, Table A3, Table A4, Table A5 and Table A6 in Appendix A and Table 8). Despite the noticeable gains in the LSTM model’s performance due to exogenous features, the ANN and LSTM-CVT models’ overachieved all the sequence-to-sequence LSTM models. 

The proposed LSTM-CVT method produced promising results as all the best performing models per prediction horizon employed this new methodology. This tendency can also be observed for each prediction horizon/window size combination presented in Table A1, Table A2, Table A3, Table A4, Table A5 and Table A6. Per each parameter combination (window size + feature ranking method + percentage), the performances of this new methodology and ANNs were compared. Results showed how the LSTM-CVT models delivered a lower average *MSE* than the ANN models in 85% of the total combinations considered. Additionally, models using this methodology performed better than sequence-to-sequence LSTMs models, especially for the *t* + 4 (*ρ* ≈ 0.70 against *ρ* ≈ 0.74) and *t* + 8 (*ρ* ≈ 0.63 against *ρ* ≈ 0.66) prediction horizons.

The percentages of lagged features selected, the feature ranking to be employed and the optimal window sizes were also discussed. Results reveal that forecasting models using a further prediction horizon need to use more information and more exogenous variables. In contrast, models for a closer prediction horizon only need the time series data and less exogenous features.

As results indicate, the new LSTM-CVT technique could be a valuable alternative to standard LSTMs and ANNs to predict NO_2_ concentrations. This novel method represents an improvement against all the other methods used, which are among the most representative in NO_2_ time series forecasting literature. Additionally, it is also important to outline the excellent performance of the exogenous models. In the case of ANN-EX and LSTM-CVT-EX models, a new methodology using feature ranking methods was also proposed to deal with the increasing lagged variables as the window sizes grow. In this approach, the importance of the selection of the more significant lagged features becomes essential. Thus, new feature selection techniques will be tested with LSTM-CVT in future works. Furthermore, it is also necessary to highlight the Bayesian optimization procedure employed to train the sequence-to-sequence LSTM models. According to a set of limits previously established, this procedure allows an automatic search of optimal hyperparameters. As a result, the chances of finding the real optimal hyperparameters are considerably higher than other approaches followed in the scientific literature.

Finally, as stated in previous sections, nitrogen dioxide plays a principal role among air pollutants due to the study area’s inherent characteristics. The proposed models and the new methodologies presented can help to predict exceedance episodes in the NO_2_ concentrations. They can act as decision-making tools that allow the governmental and environmental agencies to take the necessary measures to avoid the possible harmful effects and the associated air quality demise. Additionally, these new methodologies can be applied to other pollutants forecasting and help obtain better AQI predictions in the study area. 

## Figures and Tables

**Table 1:** List of variables included in the database.

Variable	Abbreviation	Unit	Variable Numbers
NO_2_ concentration	-	µg/m^3^	1–14
NO_x_ concentration	-	µg/m^3^	15–29
O_3_ concentration	-	µg/m^3^	30–37
SO_2_ concentration	-	µg/m^3^	38–53
Atmospheric pressure	AP	hPa	54–56
Rainfall	RA	l/m^2^	57–60
Relative humidity	RH	%	61–64
Solar radiation	SR	w/m^2^	65–67
Temperature	T	°C	68–70
Wind direction	WD	°	71–74
Wind speed	WS	km/h	75–77

**Table 2:** Monitoring and weather station codes. The pollutants or meteorological variables measured at each station are indicated. The meaning of the abbreviations used for the meteorological variables is shown in Table 1.

Code	Station	NO_2_	NO_x_	O_3_	SO_2_	AP	RA	RH	SR	T	WD	WS
1	EPS Algeciras	x	x	x	x	-	-	-	-	-	-	-
2	Campamento	x	x	x	x	-	-	-	-	-	-	-
3	Los Cortijillos	x	x	x	x	-	-	-	-	-	-	-
4	Esc. Hostelería	x	x	-	x	-	-	-	-	-	-	-
5	Col. Los Barrios	x	x	-	x	-	-	-	-	-	-	-
6	Col. Carteya	x	x	x	x	-	-	-	-	-	-	-
7	El Rinconcillo	x	x	-	x	-	-	-	-	-	-	-
8	Palmones	x	x	-	x	-	-	-	-	-	-	-
9	Est. San Roque	x	x	-	x	-	-	-	-	-	-	-
10	El Zabal	x	x	-	x	-	-	-	-	-	-	-
11	Economato	x	x	-	x	-	-	-	-	-	-	-
12	Guadarranque	x	x	x	x	-	-	-	-	-	-	-
13	La Línea	x	x	x	x	-	-	-	-	-	-	-
14	Madrevieja	x	x	-	x	-	-	-	-	-	-	-
15	Los Barrios	-	x	x	x	-	-	-	-	-	-	-
16	Alcornocales	-	-	x		-	-	-	-	-	-	-
17	Puente Mayorga	-	-	-	x	-	-	-	-	-	-	-
W1	La Línea	-	-	-	-	-	x	x	-	x	x	x
	weather station											
W2	Los Barrios	-	-	-	-	x	x	x	x	-	x	-
	weather station											
W3	Cepsa weather	-	-	-	-	x	x	x	x	-	-	-
	station (10 m)											
W4	Cepsa weather	-	-	-	-	-	-	-	-	x	x	x
	station (15 m)											
W5	Cepsa weather	-	-	-	-	x	x	x	x	x	x	x
	station (60 m)											

**Table 3:** Summary of the NO_2_ forecasting models employed in this paper. The same prediction horizons are utilized in all the cases (*t* + 1, *t* + 4, and *t* + 8). Ws indicates the window size.

Model Name	Method	Dataset	Ws	Ranking Method	%
LSTM-UN	Sequence-to-sequence LSTM	Univariate	-	-	-
LSTM-EX	Sequence-to-sequence LSTM	Exogenous	-	-	-
LSTM-CVT-UN	Sequence-to-sequence LSTM + time series cross-validation	Lagged univariate	24, 48, 72	-	-
LSTM-CVT-EX	Sequence-to-sequence LSTM + time series cross-validation	Lagged exogenous	24, 48, 72	MI, mRMR, MIC, SRC, mRMR-SRC	5, 10, 15
ANN-UN	ANN + time series cross-validation	Lagged univariate	24, 48, 72	-	-
ANN-EX	ANN + time series cross-validation	Lagged exogenous	24, 48, 72	MI, mRMR, MIC, SRC, mRMR-SRC	5, 10, 15

**Table 4:** Summary of the parameters used in the LSTM models.

Parameters	Values
LSTM neurons	1–800
Minibatch size	8, 16, 32, 64, 128, 256, 512, 1024, 2048
Initial learning rate	0.0005–0.05
L2 regularization factor	0.00005–0.0009
Dropout probability	0.0001–0.999
Gradient decay factor	0–0.999

**Table 5:** LSTM models architecture.

Layer Number	Layer Name
1	sequence input layer
2	LSTM layer
3	dropout layer
4	fully-connected layer
5	output layer (regression layer)

**Table 6:** Summary of the parameters used in the ANN models.

Parameters	Values
Neurons	1–25
Cross-validation scheme for time series	5-fold
Maximum number of epochs	2000
Max_fail (validation checks)	200

**Table 7:** Top models per prediction horizon.

Prediction Horizon	Method Name	*ws*	Feature Ranking Method	%	*ρ*	*MSE*	*d*	*MAE*	*nh*
*t* + 1	LSTM-CVT-EX	48	IM	15	0.899	97.707	0.942	6.534	580
*t* + 4	LSTM-CVT-EX	24	IM	15	0.737	231.715	0.829	10.879	507
*t* + 8	LSTM-CVT-EX	24	mRMR-SRC	15	0.659	286.364	0.769	12.683	551

**Table 8:** Percentage changes in the $\bar{MSE}$ and the $\bar{\rho }$ of the models of Table A1, Table A2, Table A3, Table A4, Table A5 and Table A6 after including exogenous input variables.

Model Comparison	t + 1		*t* + 4		*t* + 8	
	*MSE*	*ρ*	*MSE*	*ρ*	*MSE*	*ρ*
LSTM	−5.33%	1.83%	−7.23%	10.39%	−12.35%	14.16%
LSTM-CVT (24 *ws*)	−11.14%	1.70%	−16.97%	10.16%	−13.79%	12.65%
LSTM-CVT (48 *ws*)	−10.53%	1.58%	−14.86%	9.16%	−13.36%	11.90%
LSTM-CVT (72 *ws*)	−10.04%	1.47%	−14.08%	8.25%	−11.02%	10.71%
ANN (24 *ws*)	−8.64%	1.24%	−16.55%	10.26%	−12.00%	11.36%
ANN (48 *ws*)	−8.83%	1.24%	−12.37%	7.58%	−12.13%	11.07%
ANN (72 *ws*)	−8.30%	1.24%	−14.84%	8.44%	−11.40%	10.90%

## Data Availability

Not applicable.

## Appendix A

Additional tables showing the complete list of models built using univariate datasets and the top models per window size and method using exogenous datasets are presented in this appendix (Table A1, Table A2, Table A3, Table A4, Table A5 and Table A6). Additionally, the complete list of models created using the exogenous dataset is shown in Table A7, Table A8 and Table A9. In these tables, *ws* is the size of the autoregressive window, *nh* is the number of hidden units, *DP* is de dropout probability, *MBS* is the size of the minibatch, *LR* indicates the learning rate, *L*2*R* is the level 2 regularization factor and *GD* is the gradient decay factor.

**Table A1:** Results obtained using the univariate dataset and a prediction horizon of *t* + 1.

Method Name	*ws*	*ρ*	*MSE*	*d*	*MAE*	*nh*	*DP*	*MBS*	*LR*	*L*2*R*	*GD*
LSTM UN	-	0.876	117.860	0.930	7.112	21	0.009	16	0.040	0.001	0.559
LSTM-CVT-UN	24	0.884	110.485	0.935	6.775	599	0.572	1024	0.005	0.000	0.697
LSTM-CVT-UN	48	0.885	109.211	0.936	6.759	552	0.097	1024	0.006	0.001	0.543
LSTM-CVT-UN	72	0.886	108.766	0.936	6.752	507	0.190	512	0.001	0.000	0.430
ANN-UN	24	0.884	110.911	0.935	6.752	9	0.000	0	-	-	-
ANN-UN	48	0.884	110.021	0.936	6.743	8	0.000	0	-	-	-
ANN-UN	72	0.885	109.607	0.936	6.747	3	0.000	0	-	-	-

**Table A2:** Top models per window size and method using exogenous datasets and a prediction horizon of *t* + 1.

Method Name	*ws*	Feature Ranking Method	%	*ρ*	*MSE*	*d*	*MAE*	*nh*	*DP*	*MBS*	*LR*	*L*2*R*	*GD*
LSTM EX	-	-	0	0.892	111.576	0.931	6.839	188	0.222	2048	0.008	0.001	0.902
LSTM-CVT-EX	24	mRMR-SRC	10	0.899	98.177	0.941	6.556	535	0.655	512	0.001	0.000	0.813
LSTM-CVT-EX	48	IM	15	0.899	97.707	0.942	6.534	580	0.245	512	0.002	0.001	0.331
LSTM-CVT-EX	72	mRMR	5	0.899	97.849	0.941	6.529	317	0.610	512	0.001	0.001	0.771
ANN-EX	24	mRMR	15	0.895	101.330	0.941	6.654	8	-	-	-	-	-
ANN-EX	48	mRMR	10	0.895	100.304	0.941	6.646	8	-	-	-	-	-
ANN-EX	72	mRMR	5	0.896	100.509	0.941	6.613	7	-	-	-	-	-

**Table A3:** Results obtained using the univariate dataset and a prediction horizon of *t* + 4.

Method Name	*ws*	*ρ*	*MSE*	*d*	*MAE*	*nh*	*DP*	*MBS*	*LR*	*L*2*R*	*GD*
LSTM UN	-	0.635	303.161	0.739	12.749	99	0.184	512	0.048	0.000	0.404
LSTM-CVT-UN	24	0.669	279.061	0.780	11.904	544	0.650	1024	0.025	0.000	0.662
LSTM-CVT-UN	48	0.677	273.686	0.786	11.793	644	0.724	1024	0.006	0.000	0.481
LSTM-CVT-UN	72	0.679	272.495	0.785	11.833	555	0.749	1024	0.003	0.001	0.492
ANN-UN	24	0.663	282.913	0.778	11.894	9	0.000	0	-	-	-
ANN-UN	48	0.673	276.221	0.786	11.750	8	0.000	0	-	-	-
ANN-UN	72	0.675	275.401	0.786	11.770	6	0.000	0	-	-	-

**Table A4:** Top models per window size and method using exogenous datasets and a prediction horizon of *t* + 4.

Method Name	*ws*	Feature Ranking Method	%	*ρ*	*MSE*	*d*	*MAE*	*nh*	*DP*	*MBS*	*LR*	*L*2*R*	*GD*
LSTM EX	-	-	-	0.701	281.231	0.788	11.647	151	0.383	2048	0.005	0.000	0.453
LSTM-CVT-EX	24	IM	15	0.737	231.715	0.829	10.879	507	0.133	1024	0.003	0.001	0.947
LSTM-CVT-EX	48	mRMR	15	0.739	233.006	0.829	11.262	496	0.903	256	0.001	0.001	0.911
LSTM-CVT-EX	72	mRMR-SRC	15	0.735	234.117	0.825	11.246	624	0.941	128	0.001	0.000	0.665
ANN-EX	24	IM	15	0.731	236.096	0.825	10.862	7	-	-	-	-	-
ANN-EX	48	mRMR	15	0.724	242.063	0.820	11.366	3	-	-	-	-	-
ANN-EX	72	IM	10	0.732	234.520	0.827	10.945	6	-	-	-	-	-

**Table A5:** Results obtained using the univariate dataset and a prediction horizon of *t* + 8.

Method Name	*ws*	*ρ*	*MSE*	*d*	*MAE*	*nh*	*DP*	*MBS*	*LR*	*L*2*R*	*GD*
LSTM UN	-	0.558	348.821	0.683	13.953	61	0.067	1024	0.023	0.000	0.092
LSTM-CVT-UN	24	0.585	332.177	0.708	13.488	308	0.289	512	0.005	0.000	0.468
LSTM-CVT-UN	48	0.588	330.772	0.705	13.617	307	0.694	1024	0.002	0.001	0.040
LSTM-CVT-UN	72	0.588	331.065	0.710	13.566	786	0.009	1024	0.002	0.001	0.026
ANN-UN	24	0.581	334.876	0.704	13.453	5	-	-	-	-	-
ANN-UN	48	0.587	330.907	0.710	13.413	5	-	-	-	-	-
ANN-UN	72	0.587	331.104	0.710	13.433	4	-	-	-	-	-

**Table A6:** Top models per window size and method using exogenous datasets and a prediction horizon of *t* + 8.

Method Name	*ws*	Feature Ranking Method	%	*ρ*	*MSE*	*d*	*MAE*	*nh*	*DP*	*MBS*	*LR*	*L*2*R*	*GD*
LSTM EX	-	-	-	0.634	305.733	0.751	12.563	371	0.472	2048	0.007	0.000	0.920
LSTM-CVT-EX	24	mRMR-SRC	15	0.659	286.364	0.769	12.683	551	0.908	1024	0.002	0.001	0.304
LSTM-CVT-EX	48	IM	10	0.658	286.586	0.770	12.465	797	0.054	2048	0.002	0.001	0.765
LSTM-CVT-EX	72	IM	15	0.651	292.585	0.759	12.398	429	0.920	256	0.001	0.001	0.614
ANN-EX	24	mRMR-SRC	10	0.647	294.692	0.754	12.641	2	-	-	-	-	-
ANN-EX	48	IM	10	0.652	290.773	0.763	12.530	6	-	-	-	-	-
ANN-EX	72	IM	10	0.651	293.371	0.758	12.403	5	-	-	-	-	-

**Table A7:** Forecasting models using exogenous datasets and a prediction horizon of *t* + 1.

Method Name	*ws*	Feature Ranking Method	%	*ρ*	*MSE*	*d*	*MAE*	*nh*	*DP*	*MBS*	*LR*	*L*2*R*	*GD*
LSTM EX	-	-	-	0.892	111.576	0.931	6.839	188	0.222	2048	0.008	0.001	0.902
ANN-EX	24	IM	5	0.885	110.274	0.934	6.786	16	-	-	-	-	-
ANN-EX	24	IM	10	0.891	104.645	0.938	6.689	7	-	-	-	-	-
ANN-EX	24	IM	15	0.893	102.962	0.939	6.654	9	-	-	-	-	-
ANN-EX	24	mRMR	5	0.894	102.256	0.940	6.588	3	-	-	-	-	-
ANN-EX	24	mRMR	10	0.895	101.613	0.940	6.607	5	-	-	-	-	-
ANN-EX	24	mRMR	15	0.895	101.330	0.941	6.654	8	-	-	-	-	-
ANN-EX	24	MIC	5	0.890	106.048	0.937	6.679	1	-	-	-	-	-
ANN-EX	24	MIC	10	0.891	104.701	0.938	6.666	2	-	-	-	-	-
ANN-EX	24	MIC	15	0.893	103.347	0.939	6.671	4	-	-	-	-	-
ANN-EX	24	SRC	5	0.892	104.605	0.938	6.665	2	-	-	-	-	-
ANN-EX	24	SRC	10	0.892	103.781	0.938	6.687	2	-	-	-	-	-
ANN-EX	24	SRC	15	0.893	103.320	0.939	6.679	4	-	-	-	-	-
ANN-EX	24	mRMR-SRC	5	0.894	102.645	0.939	6.632	3	-	-	-	-	-
ANN-EX	24	mRMR-SRC	10	0.894	102.041	0.939	6.676	9	-	-	-	-	-
ANN-EX	24	mRMR-SRC	15	0.895	101.525	0.941	6.614	9	-	-	-	-	-
ANN-EX	48	IM	5	0.887	108.403	0.935	6.751	15	-	-	-	-	-
ANN-EX	48	IM	10	0.894	102.173	0.939	6.643	12	-	-	-	-	-
ANN-EX	48	IM	15	0.894	101.563	0.940	6.669	6	-	-	-	-	-
ANN-EX	48	mRMR	5	0.895	100.924	0.940	6.607	7	-	-	-	-	-
ANN-EX	48	mRMR	10	0.895	100.304	0.941	6.646	8	-	-	-	-	-
ANN-EX	48	mRMR	15	0.895	100.606	0.941	6.662	10	-	-	-	-	-
ANN-EX	48	MIC	5	0.845	124.345	0.898	7.232	2	-	-	-	-	-
ANN-EX	48	MIC	10	0.894	102.865	0.940	6.656	7	-	-	-	-	-
ANN-EX	48	MIC	15	0.893	102.690	0.940	6.711	6	-	-	-	-	-
ANN-EX	48	SRC	5	0.894	102.237	0.939	6.632	8	-	-	-	-	-
ANN-EX	48	SRC	10	0.894	101.918	0.940	6.672	10	-	-	-	-	-
ANN-EX	48	SRC	15	0.894	102.023	0.940	6.725	8	-	-	-	-	-
ANN-EX	48	mRMR-SRC	5	0.895	101.190	0.940	6.641	6	-	-	-	-	-
ANN-EX	48	mRMR-SRC	10	0.894	101.561	0.940	6.653	5	-	-	-	-	-
ANN-EX	48	mRMR-SRC	15	0.894	102.175	0.940	6.654	8	-	-	-	-	-
ANN-EX	72	IM	5	0.887	108.411	0.935	6.760	20	-	-	-	-	-
ANN-EX	72	IM	10	0.895	100.736	0.941	6.623	10	-	-	-	-	-
ANN-EX	72	IM	15	0.895	100.879	0.941	6.641	10	-	-	-	-	-
ANN-EX	72	mRMR	5	0.896	100.509	0.941	6.613	7	-	-	-	-	-
ANN-EX	72	mRMR	10	0.895	101.348	0.941	6.755	6	-	-	-	-	-
ANN-EX	72	mRMR	15	0.893	103.073	0.939	6.944	5	-	-	-	-	-
ANN-EX	72	MIC	5	0.893	103.387	0.939	6.680	6	-	-	-	-	-
ANN-EX	72	MIC	10	0.893	102.885	0.939	6.712	4	-	-	-	-	-
ANN-EX	72	MIC	15	0.893	103.189	0.939	6.804	5	-	-	-	-	-
ANN-EX	72	SRC	5	0.894	102.267	0.939	6.660	8	-	-	-	-	-
ANN-EX	72	SRC	10	0.894	101.985	0.940	6.729	8	-	-	-	-	-
ANN-EX	72	SRC	15	0.893	102.822	0.939	6.800	5	-	-	-	-	-
ANN-EX	72	mRMR-SRC	5	0.895	101.611	0.940	6.665	6	-	-	-	-	-
ANN-EX	72	mRMR-SRC	10	0.894	103.052	0.939	6.692	5	-	-	-	-	-
ANN-EX	72	mRMR-SRC	15	0.894	102.227	0.940	6.740	6	-	-	-	-	-
LSTM-CVT-EX	24	IM	5	0.885	110.303	0.933	6.871	350	0.493	1024	0.007	0.001	0.383
LSTM-CVT-EX	24	IM	10	0.894	101.985	0.939	6.570	538	0.025	512	0.003	0.000	0.815
LSTM-CVT-EX	24	IM	15	0.893	103.847	0.936	6.794	730	0.906	1024	0.003	0.001	0.629
LSTM-CVT-EX	24	mRMR	5	0.898	98.548	0.942	6.501	432	0.078	512	0.001	0.000	0.259
LSTM-CVT-EX	24	mRMR	10	0.898	98.833	0.941	6.548	521	0.525	512	0.001	0.000	0.527
LSTM-CVT-EX	24	mRMR	15	0.898	98.390	0.942	6.551	799	0.018	512	0.004	0.001	0.685
LSTM-CVT-EX	24	MIC	5	0.893	103.470	0.938	6.646	798	0.699	512	0.001	0.001	0.841
LSTM-CVT-EX	24	MIC	10	0.896	100.597	0.940	6.545	457	0.004	256	0.001	0.001	0.984
LSTM-CVT-EX	24	MIC	15	0.897	99.495	0.941	6.540	793	0.630	256	0.001	0.001	0.345
LSTM-CVT-EX	24	SRC	5	0.895	101.656	0.939	6.596	591	0.483	512	0.002	0.000	0.334
LSTM-CVT-EX	24	SRC	10	0.897	99.848	0.940	6.556	785	0.283	512	0.001	0.001	0.912
LSTM-CVT-EX	24	SRC	15	0.897	100.081	0.940	6.582	208	0.376	512	0.001	0.001	0.929
LSTM-CVT-EX	24	mRMR-SRC	5	0.897	100.001	0.940	6.613	128	0.401	1024	0.011	0.001	0.679
LSTM-CVT-EX	24	mRMR-SRC	10	0.899	98.177	0.941	6.556	535	0.655	512	0.001	0.000	0.813
LSTM-CVT-EX	24	mRMR-SRC	15	0.897	100.188	0.939	6.665	612	0.869	256	0.001	0.001	0.375
LSTM-CVT-EX	48	IM	5	0.888	107.710	0.935	6.750	800	0.260	1024	0.004	0.001	0.644
LSTM-CVT-EX	48	IM	10	0.897	100.005	0.940	6.582	721	0.260	512	0.012	0.001	0.760
LSTM-CVT-EX	48	IM	15	0.899	97.707	0.942	6.534	580	0.245	512	0.002	0.001	0.331
LSTM-CVT-EX	48	mRMR	5	0.898	98.475	0.941	6.554	794	0.778	512	0.001	0.000	0.189
LSTM-CVT-EX	48	mRMR	10	0.897	99.701	0.940	6.688	249	0.490	1024	0.001	0.001	0.862
LSTM-CVT-EX	48	mRMR	15	0.899	97.971	0.942	6.591	628	0.734	512	0.001	0.001	0.644
LSTM-CVT-EX	48	MIC	5	0.896	100.749	0.940	6.586	721	0.260	512	0.012	0.001	0.760
LSTM-CVT-EX	48	MIC	10	0.895	103.867	0.935	6.771	736	0.921	512	0.002	0.001	0.912
LSTM-CVT-EX	48	MIC	15	0.896	102.641	0.938	6.700	450	0.608	1024	0.001	0.000	0.591
LSTM-CVT-EX	48	SRC	5	0.897	99.777	0.940	6.568	797	0.589	512	0.001	0.001	0.488
LSTM-CVT-EX	48	SRC	10	0.898	98.370	0.942	6.533	545	0.218	512	0.008	0.001	0.819
LSTM-CVT-EX	48	SRC	15	0.896	100.374	0.940	6.649	784	0.012	1024	0.001	0.001	0.913
LSTM-CVT-EX	48	mRMR-SRC	5	0.897	100.729	0.939	6.767	430	0.517	512	0.001	0.000	0.827
LSTM-CVT-EX	48	mRMR-SRC	10	0.899	97.881	0.942	6.523	721	0.260	512	0.012	0.001	0.760
LSTM-CVT-EX	48	mRMR-SRC	15	0.896	100.753	0.941	6.622	557	0.044	256	0.001	0.001	0.996
LSTM-CVT-EX	72	IM	5	0.887	108.459	0.934	6.808	783	0.327	1024	0.002	0.001	0.515
LSTM-CVT-EX	72	IM	10	0.898	98.477	0.942	6.588	796	0.478	512	0.001	0.000	0.743
LSTM-CVT-EX	72	IM	15	0.896	102.468	0.937	6.812	751	0.868	1024	0.002	0.001	0.510
LSTM-CVT-EX	72	mRMR	5	0.899	97.849	0.941	6.529	317	0.610	512	0.001	0.001	0.771
LSTM-CVT-EX	72	mRMR	10	0.897	98.863	0.941	6.688	799	0.678	512	0.001	0.001	0.447
LSTM-CVT-EX	72	mRMR	15	0.892	104.896	0.936	7.050	524	0.815	1024	0.001	0.001	0.909
LSTM-CVT-EX	72	MIC	5	0.898	99.385	0.940	6.528	428	0.523	512	0.001	0.001	0.959
LSTM-CVT-EX	72	MIC	10	0.892	106.218	0.934	6.977	795	0.008	1024	0.001	0.000	0.615
LSTM-CVT-EX	72	MIC	15	0.896	101.962	0.938	6.750	779	0.682	512	0.001	0.001	0.471
LSTM-CVT-EX	72	SRC	5	0.897	99.335	0.941	6.533	573	0.590	512	0.001	0.000	0.878
LSTM-CVT-EX	72	SRC	10	0.897	99.067	0.941	6.568	550	0.678	256	0.001	0.001	0.955
LSTM-CVT-EX	72	SRC	15	0.897	100.383	0.939	6.723	800	0.624	512	0.003	0.001	0.874
LSTM-CVT-EX	72	mRMR-SRC	5	0.899	98.050	0.941	6.518	775	0.615	512	0.005	0.001	0.643
LSTM-CVT-EX	72	mRMR-SRC	10	0.898	99.317	0.941	6.532	593	0.629	256	0.001	0.001	0.776
LSTM-CVT-EX	72	mRMR-SRC	15	0.893	105.562	0.935	6.871	771	0.902	512	0.001	0.001	0.586

**Table A8:** Forecasting models using exogenous datasets and a prediction horizon of *t* + 4.

Method Name	*ws*	Feature Ranking Method	%	*ρ*	*MSE*	*d*	*MAE*	*nh*	*DP*	*MBS*	*LR*	*L*2*R*	*GD*
LSTM EX	-	-	-	0.701	281.231	0.788	11.647	151	0.383	2048	0.005	0.000	0.453
ANN-EX	24	IM	5	0.660	285.492	0.769	12.101	19	-	-	-	-	-
ANN-EX	24	IM	10	0.699	260.018	0.793	11.481	6	-	-	-	-	-
ANN-EX	24	IM	15	0.731	236.096	0.825	10.862	7	-	-	-	-	-
ANN-EX	24	mRMR	5	0.712	249.692	0.807	11.307	5	-	-	-	-	-
ANN-EX	24	mRMR	10	0.719	244.933	0.813	11.243	3	-	-	-	-	-
ANN-EX	24	mRMR	15	0.720	244.375	0.817	11.327	4	-	-	-	-	-
ANN-EX	24	MIC	5	0.692	265.108	0.789	11.546	4	-	-	-	-	-
ANN-EX	24	MIC	10	0.709	252.427	0.806	11.225	3	-	-	-	-	-
ANN-EX	24	MIC	15	0.705	258.000	0.798	11.237	3	-	-	-	-	-
ANN-EX	24	SRC	5	0.695	262.726	0.790	11.593	3	-	-	-	-	-
ANN-EX	24	SRC	10	0.706	254.125	0.803	11.410	3	-	-	-	-	-
ANN-EX	24	SRC	15	0.713	249.092	0.813	11.168	5	-	-	-	-	-
ANN-EX	24	mRMR-SRC	5	0.702	257.063	0.798	11.464	3	-	-	-	-	-
ANN-EX	24	mRMR-SRC	10	0.704	256.119	0.798	11.578	2	-	-	-	-	-
ANN-EX	24	mRMR-SRC	15	0.711	251.320	0.807	11.203	2	-	-	-	-	-
ANN-EX	48	IM	5	0.661	284.350	0.770	12.126	18	-	-	-	-	-
ANN-EX	48	IM	10	0.721	243.186	0.814	11.141	3	-	-	-	-	-
ANN-EX	48	IM	15	0.731	237.550	0.824	10.827	4	-	-	-	-	-
ANN-EX	48	mRMR	5	0.716	246.936	0.810	11.300	3	-	-	-	-	-
ANN-EX	48	mRMR	10	0.723	242.648	0.819	11.342	3	-	-	-	-	-
ANN-EX	48	mRMR	15	0.724	242.063	0.820	11.366	3	-	-	-	-	-
ANN-EX	48	MIC	5	0.708	253.114	0.805	11.254	3	-	-	-	-	-
ANN-EX	48	MIC	10	0.711	252.296	0.806	11.139	2	-	-	-	-	-
ANN-EX	48	MIC	15	0.715	249.283	0.809	11.139	3	-	-	-	-	-
ANN-EX	48	SRC	5	0.705	254.645	0.802	11.379	4	-	-	-	-	-
ANN-EX	48	SRC	10	0.713	248.973	0.810	11.368	3	-	-	-	-	-
ANN-EX	48	SRC	15	0.719	244.905	0.816	11.319	4	-	-	-	-	-
ANN-EX	48	mRMR-SRC	5	0.680	262.661	0.773	11.737	3	-	-	-	-	-
ANN-EX	48	mRMR-SRC	10	0.721	243.114	0.820	11.016	4	-	-	-	-	-
ANN-EX	48	mRMR-SRC	15	0.722	242.069	0.821	11.135	4	-	-	-	-	-
ANN-EX	72	IM	5	0.657	287.445	0.766	12.230	15	-	-	-	-	-
ANN-EX	72	IM	10	0.732	234.520	0.827	10.945	6	-	-	-	-	-
ANN-EX	72	IM	15	0.725	240.807	0.822	10.965	4	-	-	-	-	-
ANN-EX	72	mRMR	5	0.721	242.891	0.817	11.252	4	-	-	-	-	-
ANN-EX	72	mRMR	10	0.725	242.642	0.824	11.439	3	-	-	-	-	-
ANN-EX	72	mRMR	15	0.719	247.925	0.816	11.684	2	-	-	-	-	-
ANN-EX	72	MIC	5	0.709	255.310	0.803	11.138	3	-	-	-	-	-
ANN-EX	72	MIC	10	0.678	263.067	0.773	11.534	2	-	-	-	-	-
ANN-EX	72	MIC	15	0.719	245.159	0.813	11.324	2	-	-	-	-	-
ANN-EX	72	SRC	5	0.709	252.163	0.806	11.351	3	-	-	-	-	-
ANN-EX	72	SRC	10	0.718	245.538	0.816	11.331	4	-	-	-	-	-
ANN-EX	72	SRC	15	0.718	245.315	0.816	11.331	4	-	-	-	-	-
ANN-EX	72	mRMR-SRC	5	0.715	247.544	0.813	11.358	3	-	-	-	-	-
ANN-EX	72	mRMR-SRC	10	0.719	244.652	0.817	11.187	3	-	-	-	-	-
ANN-EX	72	mRMR-SRC	15	0.721	243.525	0.817	11.317	2	-	-	-	-	-
LSTM-CVT-EX	24	IM	5	0.665	282.079	0.775	12.069	239	0.019	1024	0.039	0.001	0.854
LSTM-CVT-EX	24	IM	10	0.701	257.806	0.799	11.497	445	0.162	512	0.022	0.001	0.289
LSTM-CVT-EX	24	IM	15	0.737	231.715	0.829	10.879	507	0.133	1024	0.003	0.001	0.947
LSTM-CVT-EX	24	mRMR	5	0.723	241.701	0.822	11.129	418	0.044	1024	0.005	0.000	0.248
LSTM-CVT-EX	24	mRMR	10	0.731	237.060	0.822	11.248	791	0.863	2048	0.001	0.000	0.437
LSTM-CVT-EX	24	mRMR	15	0.734	236.293	0.823	11.354	439	0.715	2048	0.047	0.001	0.300
LSTM-CVT-EX	24	MIC	5	0.697	261.007	0.792	11.570	659	0.526	1024	0.001	0.000	0.481
LSTM-CVT-EX	24	MIC	10	0.720	244.538	0.811	11.164	239	0.697	1024	0.002	0.001	0.864
LSTM-CVT-EX	24	MIC	15	0.715	251.915	0.796	11.261	366	0.941	1024	0.001	0.000	0.736
LSTM-CVT-EX	24	SRC	5	0.706	255.624	0.795	11.670	144	0.792	512	0.002	0.000	0.200
LSTM-CVT-EX	24	SRC	10	0.713	248.940	0.809	11.424	792	0.456	2048	0.001	0.001	0.255
LSTM-CVT-EX	24	SRC	15	0.725	239.376	0.826	10.971	499	0.400	1024	0.002	0.000	0.966
LSTM-CVT-EX	24	mRMR-SRC	5	0.721	244.194	0.812	11.339	263	0.582	1024	0.049	0.000	0.741
LSTM-CVT-EX	24	mRMR-SRC	10	0.725	242.032	0.820	11.362	305	0.638	1024	0.001	0.001	0.346
LSTM-CVT-EX	24	mRMR-SRC	15	0.731	235.361	0.829	10.912	548	0.734	2048	0.002	0.000	0.760
LSTM-CVT-EX	48	IM	5	0.669	279.187	0.781	11.986	800	0.001	512	0.013	0.001	0.862
LSTM-CVT-EX	48	IM	10	0.735	233.473	0.823	10.971	800	0.878	2048	0.002	0.001	0.180
LSTM-CVT-EX	48	IM	15	0.736	235.097	0.815	11.024	691	0.922	1024	0.018	0.001	0.657
LSTM-CVT-EX	48	mRMR	5	0.731	236.314	0.822	11.104	303	0.758	2048	0.009	0.001	0.473
LSTM-CVT-EX	48	mRMR	10	0.734	235.490	0.830	11.209	784	0.496	2048	0.001	0.001	0.347
LSTM-CVT-EX	48	mRMR	15	0.739	233.006	0.829	11.262	496	0.903	256	0.001	0.001	0.911
LSTM-CVT-EX	48	MIC	5	0.717	247.414	0.805	11.298	200	0.755	1024	0.001	0.001	0.837
LSTM-CVT-EX	48	MIC	10	0.712	252.686	0.794	11.469	667	0.977	1024	0.001	0.000	0.460
LSTM-CVT-EX	48	MIC	15	0.718	250.441	0.800	11.168	698	0.970	1024	0.001	0.000	0.414
LSTM-CVT-EX	48	SRC	5	0.717	245.840	0.813	11.294	519	0.776	1024	0.002	0.000	0.964
LSTM-CVT-EX	48	SRC	10	0.725	240.348	0.819	11.164	798	0.876	1024	0.001	0.001	0.390
LSTM-CVT-EX	48	SRC	15	0.727	240.581	0.816	11.323	591	0.940	512	0.001	0.000	0.582
LSTM-CVT-EX	48	mRMR-SRC	5	0.722	243.729	0.814	11.411	514	0.878	2048	0.002	0.000	0.784
LSTM-CVT-EX	48	mRMR-SRC	10	0.734	234.106	0.825	11.020	798	0.889	512	0.002	0.000	0.978
LSTM-CVT-EX	48	mRMR-SRC	15	0.734	234.150	0.824	11.122	799	0.930	256	0.002	0.000	0.619
LSTM-CVT-EX	72	IM	5	0.660	285.061	0.770	12.237	225	0.155	1024	0.030	0.001	0.341
LSTM-CVT-EX	72	IM	10	0.734	234.785	0.822	11.120	722	0.892	2048	0.001	0.001	0.058
LSTM-CVT-EX	72	IM	15	0.731	236.581	0.823	11.371	799	0.744	2048	0.001	0.000	0.938
LSTM-CVT-EX	72	mRMR	5	0.732	236.074	0.822	11.157	425	0.877	2048	0.001	0.000	0.138
LSTM-CVT-EX	72	mRMR	10	0.726	244.361	0.809	11.737	530	0.959	1024	0.001	0.000	0.628
LSTM-CVT-EX	72	mRMR	15	0.733	241.713	0.824	11.720	391	0.936	256	0.001	0.000	0.365
LSTM-CVT-EX	72	MIC	5	0.711	255.384	0.793	11.304	315	0.921	2048	0.001	0.000	0.750
LSTM-CVT-EX	72	MIC	10	0.723	247.852	0.804	11.057	219	0.889	2048	0.001	0.000	0.548
LSTM-CVT-EX	72	MIC	15	0.721	247.288	0.807	11.168	797	0.972	1024	0.001	0.000	0.629
LSTM-CVT-EX	72	SRC	5	0.721	243.729	0.814	11.246	333	0.814	2048	0.001	0.000	0.498
LSTM-CVT-EX	72	SRC	10	0.728	238.752	0.821	11.206	794	0.836	2048	0.001	0.000	0.253
LSTM-CVT-EX	72	SRC	15	0.730	241.677	0.806	11.550	391	0.941	512	0.001	0.001	0.335
LSTM-CVT-EX	72	mRMR-SRC	5	0.729	240.217	0.818	11.459	444	0.908	1024	0.006	0.000	0.205
LSTM-CVT-EX	72	mRMR-SRC	10	0.725	242.822	0.808	11.344	569	0.945	2048	0.017	0.001	0.030
LSTM-CVT-EX	72	mRMR-SRC	15	0.735	234.117	0.825	11.246	624	0.941	128	0.001	0.000	0.665

**Table A9:** Forecasting models using exogenous datasets and a prediction horizon of *t* + 8.

Method Name	*ws*	Feature Ranking Method	%	*ρ*	*MSE*	*d*	*MAE*	*nh*	*DP*	*MBS*	*LR*	*L*2*R*	*GD*
LSTM EX	-	-	-	0.637	305.733	0.751	12.563	371	0.472	2048	0.007	0.000	0.920
ANN-EX	24	IM	5	0.550	352.356	0.677	13.939	3	-	-	-	-	-
ANN-EX	24	IM	10	0.637	300.655	0.745	12.932	4	-	-	-	-	-
ANN-EX	24	IM	15	0.645	294.947	0.757	12.738	4	-	-	-	-	-
ANN-EX	24	mRMR	5	0.635	303.618	0.748	13.143	3	-	-	-	-	-
ANN-EX	24	mRMR	10	0.641	300.618	0.749	13.172	2	-	-	-	-	-
ANN-EX	24	mRMR	15	0.639	301.988	0.748	13.242	2	-	-	-	-	-
ANN-EX	24	MIC	5	0.607	319.848	0.716	13.308	1	-	-	-	-	-
ANN-EX	24	MIC	10	0.622	310.245	0.734	12.954	3	-	-	-	-	-
ANN-EX	24	MIC	15	0.621	310.650	0.735	12.854	2	-	-	-	-	-
ANN-EX	24	SRC	5	0.619	312.867	0.729	13.266	3	-	-	-	-	-
ANN-EX	24	SRC	10	0.623	309.851	0.737	13.127	2	-	-	-	-	-
ANN-EX	24	SRC	15	0.626	307.853	0.745	13.074	3	-	-	-	-	-
ANN-EX	24	mRMR-SRC	5	0.625	309.607	0.733	12.761	2	-	-	-	-	-
ANN-EX	24	mRMR-SRC	10	0.647	294.692	0.754	12.641	2	-	-	-	-	-
ANN-EX	24	mRMR-SRC	15	0.645	295.833	0.753	12.743	2	-	-	-	-	-
ANN-EX	48	IM	5	0.573	339.662	0.690	13.796	12	-	-	-	-	-
ANN-EX	48	IM	10	0.652	290.773	0.763	12.530	6	-	-	-	-	-
ANN-EX	48	IM	15	0.637	300.806	0.749	12.679	3	-	-	-	-	-
ANN-EX	48	mRMR	5	0.648	295.927	0.753	13.043	3	-	-	-	-	-
ANN-EX	48	mRMR	10	0.607	313.362	0.721	13.649	2	-	-	-	-	-
ANN-EX	48	mRMR	15	0.638	304.727	0.748	13.390	2	-	-	-	-	-
ANN-EX	48	MIC	5	0.625	308.300	0.735	12.952	3	-	-	-	-	-
ANN-EX	48	MIC	10	0.628	306.967	0.738	12.813	2	-	-	-	-	-
ANN-EX	48	MIC	15	0.631	304.971	0.737	12.944	2	-	-	-	-	-
ANN-EX	48	SRC	5	0.609	318.518	0.722	13.440	1	-	-	-	-	-
ANN-EX	48	SRC	10	0.625	308.162	0.738	13.074	2	-	-	-	-	-
ANN-EX	48	SRC	15	0.626	310.616	0.741	13.305	4	-	-	-	-	-
ANN-EX	48	mRMR-SRC	5	0.642	297.816	0.754	12.607	3	-	-	-	-	-
ANN-EX	48	mRMR-SRC	10	0.642	297.925	0.748	12.675	2	-	-	-	-	-
ANN-EX	48	mRMR-SRC	15	0.641	298.244	0.748	12.821	2	-	-	-	-	-
ANN-EX	72	IM	5	0.571	340.793	0.689	13.842	11	-	-	-	-	-
ANN-EX	72	IM	10	0.651	293.371	0.758	12.403	5	-	-	-	-	-
ANN-EX	72	IM	15	0.638	300.241	0.751	12.685	3	-	-	-	-	-
ANN-EX	72	mRMR	5	0.636	306.962	0.748	13.471	2	-	-	-	-	-
ANN-EX	72	mRMR	10	0.636	309.168	0.752	13.553	2	-	-	-	-	-
ANN-EX	72	mRMR	15	0.599	319.068	0.708	13.782	1	-	-	-	-	-
ANN-EX	72	MIC	5	0.614	315.956	0.722	13.032	1	-	-	-	-	-
ANN-EX	72	MIC	10	0.628	307.048	0.733	12.990	2	-	-	-	-	-
ANN-EX	72	MIC	15	0.643	297.249	0.753	12.938	2	-	-	-	-	-
ANN-EX	72	SRC	5	0.620	311.599	0.734	13.192	2	-	-	-	-	-
ANN-EX	72	SRC	10	0.627	309.933	0.742	13.264	4	-	-	-	-	-
ANN-EX	72	SRC	15	0.640	303.055	0.749	13.346	2	-	-	-	-	-
ANN-EX	72	mRMR-SRC	5	0.635	302.719	0.741	12.782	2	-	-	-	-	-
ANN-EX	72	mRMR-SRC	10	0.639	299.470	0.748	12.761	2	-	-	-	-	-
ANN-EX	72	mRMR-SRC	15	0.613	308.235	0.724	13.297	2	-	-	-	-	-
LSTM-CVT-EX	24	IM	5	0.550	352.459	0.674	14.149	28	0.471	256	0.001	0.001	0.008
LSTM-CVT-EX	24	IM	10	0.640	298.837	0.751	12.914	706	0.053	512	0.002	0.001	0.014
LSTM-CVT-EX	24	IM	15	0.654	290.565	0.757	12.747	260	0.817	2048	0.002	0.000	0.007
LSTM-CVT-EX	24	mRMR	5	0.649	296.467	0.756	13.137	72	0.772	1024	0.003	0.000	0.236
LSTM-CVT-EX	24	mRMR	10	0.656	296.280	0.749	13.355	173	0.903	256	0.001	0.000	0.250
LSTM-CVT-EX	24	mRMR	15	0.662	294.354	0.752	13.397	355	0.935	256	0.003	0.000	0.534
LSTM-CVT-EX	24	MIC	5	0.629	307.538	0.728	13.158	157	0.760	1024	0.001	0.001	0.189
LSTM-CVT-EX	24	MIC	10	0.638	300.273	0.745	12.902	799	0.754	1024	0.005	0.000	0.370
LSTM-CVT-EX	24	MIC	15	0.640	301.889	0.735	13.190	446	0.898	2048	0.002	0.001	0.219
LSTM-CVT-EX	24	SRC	5	0.624	310.983	0.732	13.411	598	0.685	512	0.008	0.001	0.603
LSTM-CVT-EX	24	SRC	10	0.631	306.161	0.743	13.218	207	0.620	2048	0.001	0.000	0.695
LSTM-CVT-EX	24	SRC	15	0.636	301.818	0.753	12.983	795	0.739	2048	0.003	0.001	0.330
LSTM-CVT-EX	24	mRMR-SRC	5	0.615	318.469	0.736	13.213	556	0.747	1024	0.025	0.001	0.002
LSTM-CVT-EX	24	mRMR-SRC	10	0.656	290.865	0.756	12.889	143	0.721	64	0.002	0.001	0.761
LSTM-CVT-EX	24	mRMR-SRC	15	0.659	286.364	0.769	12.683	551	0.908	1024	0.002	0.001	0.304
LSTM-CVT-EX	48	IM	5	0.572	342.479	0.679	14.167	150	0.887	2048	0.050	0.001	0.771
LSTM-CVT-EX	48	IM	10	0.658	286.586	0.770	12.465	797	0.054	2048	0.002	0.001	0.765
LSTM-CVT-EX	48	IM	15	0.634	302.417	0.745	12.833	339	0.966	2048	0.001	0.000	0.936
LSTM-CVT-EX	48	mRMR	5	0.658	294.126	0.764	13.153	332	0.864	512	0.004	0.001	0.695
LSTM-CVT-EX	48	mRMR	10	0.652	299.662	0.746	13.437	796	0.956	128	0.001	0.001	0.698
LSTM-CVT-EX	48	mRMR	15	0.651	299.747	0.750	13.436	791	0.954	2048	0.001	0.001	0.454
LSTM-CVT-EX	48	MIC	5	0.636	302.669	0.736	13.030	341	0.851	256	0.001	0.001	0.278
LSTM-CVT-EX	48	MIC	10	0.638	302.759	0.731	13.129	658	0.969	1024	0.001	0.000	0.328
LSTM-CVT-EX	48	MIC	15	0.637	304.721	0.721	13.166	743	0.982	512	0.001	0.000	0.321
LSTM-CVT-EX	48	SRC	5	0.633	304.678	0.750	13.173	730	0.751	2048	0.001	0.000	0.916
LSTM-CVT-EX	48	SRC	10	0.638	302.462	0.737	13.115	775	0.953	128	0.001	0.001	0.875
LSTM-CVT-EX	48	SRC	15	0.645	298.788	0.749	13.133	376	0.872	2048	0.002	0.000	0.647
LSTM-CVT-EX	48	mRMR-SRC	5	0.654	291.138	0.765	12.810	475	0.674	1024	0.041	0.001	0.359
LSTM-CVT-EX	48	mRMR-SRC	10	0.654	291.135	0.750	12.790	629	0.938	1024	0.030	0.001	0.775
LSTM-CVT-EX	48	mRMR-SRC	15	0.647	295.582	0.748	12.937	414	0.969	2048	0.002	0.001	0.027
LSTM-CVT-EX	72	IM	5	0.569	345.096	0.668	14.291	153	0.939	2048	0.023	0.001	0.828
LSTM-CVT-EX	72	IM	10	0.648	295.067	0.747	12.630	503	0.949	2048	0.001	0.000	0.374
LSTM-CVT-EX	72	IM	15	0.651	292.585	0.759	12.398	429	0.920	256	0.001	0.001	0.614
LSTM-CVT-EX	72	mRMR	5	0.646	301.021	0.743	13.396	724	0.963	1024	0.003	0.001	0.434
LSTM-CVT-EX	72	mRMR	10	0.646	304.551	0.742	13.641	785	0.970	1024	0.003	0.001	0.918
LSTM-CVT-EX	72	mRMR	15	0.645	303.958	0.742	13.588	631	0.970	2048	0.001	0.000	0.634
LSTM-CVT-EX	72	MIC	5	0.627	313.017	0.706	13.478	209	0.963	512	0.001	0.000	0.413
LSTM-CVT-EX	72	MIC	10	0.639	305.979	0.713	13.223	187	0.958	512	0.001	0.001	0.135
LSTM-CVT-EX	72	MIC	15	0.635	306.077	0.720	13.155	713	0.989	512	0.001	0.001	0.072
LSTM-CVT-EX	72	SRC	5	0.633	304.459	0.743	13.133	526	0.903	2048	0.001	0.000	0.293
LSTM-CVT-EX	72	SRC	10	0.638	307.399	0.724	13.541	795	0.977	512	0.002	0.001	0.002
LSTM-CVT-EX	72	SRC	15	0.652	301.901	0.742	13.634	336	0.947	512	0.001	0.000	0.360
LSTM-CVT-EX	72	mRMR-SRC	5	0.639	300.199	0.743	12.971	580	0.962	2048	0.002	0.000	0.206
LSTM-CVT-EX	72	mRMR-SRC	10	0.650	295.055	0.751	13.037	567	0.953	512	0.001	0.000	0.172
LSTM-CVT-EX	72	mRMR-SRC	15	0.645	300.661	0.769	13.204	326	0.894	2048	0.001	0.001	0.964

**Algorithm A1.** Minimum-Redundancy-Maximum-Relevance for regression.			
	INPUT:	candidateFeatures // set of features to be ranked.	
		*Y* // target variable.	
	OUTPUT:	rankedFfeatures // features ranked	
1:	**for** feature *i* in candidateFeatures **do**		
2:		relevance = MI (*i*, *Y*);	
3:		redundancy = 0;	
4:		**for** feature *j* in candidateFeatures **do**	
5:			redundancy = redundancy + MI (*i,j*);
6:		**end for**	
7:		mrmrValues[ *i* ] = relevance − redundancy;	
8:	**end for**		
9:	rankedFeatures = sort(mrmrValues);		

**Algorithm A2.** Minimum-Redundancy-Maximum-Relevance for regression using the Spearman’s rank correlation.			
	INPUT:	candidateFeatures // set of features to be ranked.	
		*Y* // target variable.	
	OUTPUT:	rankedFfeatures // features ranked	
1:	**for** feature *i* in candidateFeatures **do**		
2:		relevance = *r* (*i*, *Y*);	
3:		redundancy = 0;	
4:		**for** feature *j* in candidateFeatures **do**	
5:			redundancy = redundancy + *r* (*i,j*);
6:		**end for**	
7:		mrmrValues[ *i* ] = relevance − redundancy;	
8:	**end for**		
9:	rankedFeatures = sort(mrmrValues);		
